# Innovative isotopic modeling and risk assessment of PTEs and PAHs in urban soils

**DOI:** 10.1038/s41598-025-22183-z

**Published:** 2025-11-03

**Authors:** Pegah Parchami, Sima Sabzalipour, Ahad Nazarpour, Maryam Mohammadi Rozbahani, Navid Ghanavati

**Affiliations:** 1 Department of Environmental Sciences, Ahv.C, Islamic Azad University, Ahvaz, Iran; 2 Department of Geology, Ahv.C, Islamic Azad University, Ahvaz, Iran; 3 Department of Soil Sciences, Ahv.C, Islamic Azad University, Ahvaz, Iran

**Keywords:** PTEs, Polycyclic aromatic hydrocarbons (PAHs), Urban soil, Health risk assessment, Innovative isotopic modeling, Lead contamination, Environmental sciences, Environmental chemistry, Environmental impact

## Abstract

This study investigates the health and environmental risks associated with Potentially Toxic Elements (PTEs) and polycyclic aromatic hydrocarbons (PAHs) in urban soils of Sanandaj City, Iran, using an innovative isotopic modeling approach. A total of 53 surface soil samples were systematically collected, revealing elevated concentrations of Zinc (Zn), Copper (Cu), Nickel (Ni), Cadmium (Cd), Chromium (Cr), Arsenic (As), and Lead (Pb), with Cd posing a significant ecological risk. Ecological risk assessments indicated low risk for most elements, except for Cd, which showed a notable environmental threat. Non-carcinogenic risk assessments, based on ingestion, inhalation, and dermal exposure, revealed no significant risks for both children and adults, although children exhibited higher risks, particularly in ingestion. However, carcinogenic risk assessments demonstrated that metals like Cr, Ni, Pb, Cd, and As exceeded the acceptable risk thresholds for both populations, highlighting a severe health concern. PAH concentrations ranged from 126.44 to 2460.87 µg/kg, with a dominance of high molecular weight PAHs, suggesting significant pyrogenic sources. A novel isotopic modeling method, based on ^206^Pb/^207^Pb and ^208^Pb/^207^Pb ratios, effectively traced lead contamination sources, identifying industrial emissions and vehicular exhaust as the primary contributors. This study offers valuable insights into urban soil contamination and provides a robust framework for environmental management and policy-making to mitigate risks from PTEs and PAHs.

**Supplementary Information:**

The online version contains supplementary material available at 10.1038/s41598-025-22183-z.

## Introduction

Urban soil pollution by potentially toxic elements (PTEs) and polycyclic aromatic hydrocarbons (PAHs) is a major environmental issue, particularly in industrialized and densely populated areas^1,2^. This pollution not only impacts human health but also harms natural ecosystems^3,4^. Sanandaj, an industrial city in western Iran, faces significant soil contamination issues, primarily due to industrial activities and traffic emissions. This study aims to evaluate the contamination of urban soils in Sanandaj by PTEs and PAHs using innovative isotopic modeling and to assess the associated health and environmental risks^5^.

Urban surface soils are considered one of the primary reservoirs for potentially toxic metals (PTMs) and other pollutants in urban environments^6,7^. Research conducted on urban soils in diverse regions around the world, including Russia^8^, India^9^, Poland^10^, and China^7^, has revealed substantial contamination of these soils with numerous toxic substances, such as PTEs and polycyclic aromatic hydrocarbons (PAHs). This soil pollution encompasses the presence of potentially toxic elements (PTEs), including PTEs and persistent organic pollutants (POPs)^11,12^. Polycyclic aromatic hydrocarbons (PAHs), petroleum-derived compounds with multi-ring aromatic structures, are significant soil pollutants due to their water insolubility, high toxicity, and carcinogenic properties, particularly for high molecular weight (HMW) PAHs, classified as persistent toxic substances by the U.S. EPA and UNEP^13–16^^17–21^.

This classification acknowledges these substances’ persistent, potentially toxic, mutagenic, and carcinogenic properties, highlighting the urgency of addressing their widespread distribution in natural environments, including soils and dust^6,7^. Understanding the intricate relationship between soil pollution and public health necessitates an exploration of human exposure pathways^22^. Inhalation, ingestion, and dermal contact are the main routes through which people encounter potentially toxic metals in soil^19,23^. The interconnectedness of these pathways emphasizes the need for a holistic approach to assessing and mitigating the health risks associated with soil pollution^22,24^. Soil contamination has significant and diverse impacts on human health. The occurrence of respiratory and cardiovascular diseases, along with an increased likelihood of cancer and other health conditions, highlights the critical need to address soil pollution^25,26^. The intricacies of these health impacts necessitate a robust understanding of the toxicological aspects and a commitment to developing effective public health interventions^27^. Mitigating urban soil pollution requires a holistic approach, integrating sustainable practices like green infrastructure, brownfield revitalization, and contaminated land remediation with stringent monitoring and evidence-based regulatory frameworks to balance human activities and environmental health^1,28^.

The global challenge of urban soil pollution demands a comprehensive and collaborative approach. From the detailed analysis of pollutants like PAHs to recognizing their regulatory implications, understanding the multifaceted dimensions of soil pollution is crucial. Human health impacts and mitigation strategies underscore the need for ongoing research, stringent monitoring, and a commitment to sustainable practices^29,30^. By embracing environmental stewardship and fostering collective responsibility for the well-being of our urban ecosystems, we can strive towards a future where soil pollution is mitigated and the delicate balance between human activities and environmental health is preserved^31,32^.

This research is designed to identify and quantify the primary sources of lead contamination in urban soils of Sanandaj, utilizing ^206^Pb/^207^Pb and ^208^Pb/^207^Pb isotopic ratios to distinguish between geogenic and anthropogenic sources^33,34^. Additionally, the study will perform a rigorous ecological risk assessment by employing established risk indices (RI) to evaluate the potential environmental impacts of PTEs in the soil, specifically focusing on their toxicity and bioavailability. In the realm of human health, the study will assess non-carcinogenic risks associated with exposure to PTEs via various exposure routes, including ingestion, inhalation, and dermal absorption, using the Hazard Index (HI) method. Furthermore, the carcinogenic risk assessment will focus on metals with known oncogenic potential, including chromium, nickel, lead, cadmium, and arsenic, estimating the cumulative cancer risk for both adult and child populations.

This study will also facilitate a comparative analysis of soil contamination levels in Sanandaj with other urban centers worldwide, with a focus on identifying the specific industrial and vehicular pollution sources unique to the region. Based on the comprehensive findings, the study will propose evidence-based environmental management frameworks and regulatory policies aimed at minimizing PTE and PAH pollution, thereby enhancing public health and promoting ecological resilience in urban soils.

## Materials and methods

### Study area

**Fig. 1:**
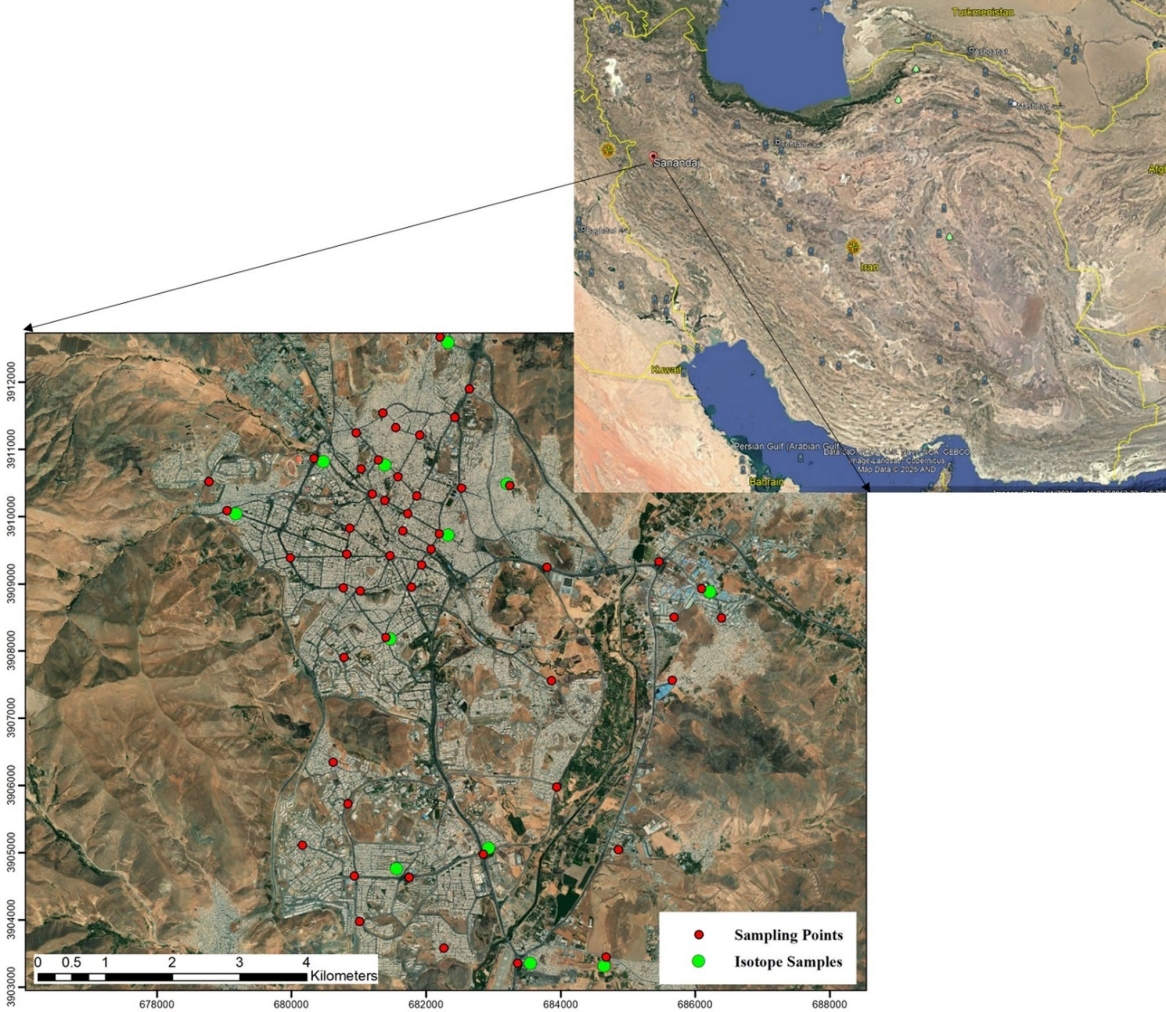
Map of Iran, Sanandaj City, and the spatial distribution of soil and isotope samples within the study area. The satellite imagery was obtained from Google Earth (Image: Google, Landsat/Copernicus). The map created using QGIS, version 3.18; https://www.qgis.org/, (Sampling Points: Red = Soil, Green = Isotope).

Sanandaj, the capital of Kurdistan Province in northwest Iran, spans approximately 3,686 km² between 35°32′N latitude and 46°18′E to 47°16′E longitude, at elevations of 1,450–1,538 m above sea level (Fig. 1)^35^. The location coordinates of the Sanandaj region in Iran are presented in Fig. 1. With a population of about 450,000, the city features diverse land uses and soils, predominantly Entisols and Inceptisols with loamy and sandy loam textures, characterized by high salinity and sodium content. Geologically, it lies within the Sanandaj-Sirjan Zone, comprising metamorphic (e.g., schist, phyllite) and sedimentary (e.g., limestone, shale) formations that influence background PTE concentrations. The region experiences an annual precipitation of 427 mm and an average temperature of 14.7 °C, with a dual climate of cold mountainous and hot arid conditions, fostering diverse vegetation that impacts soil organic matter and pollutant retention^35^.

### Collection and preparation of soil samples

Accurately assessing PTEs concentrations in soil begins with meticulous sample collection and preparation. In this study, 53 surface soil samples were systematically collected from various locations within Sanandaj City. These 53 sampling points were selected to represent diverse land uses (industrial, residential, traffic-heavy, and green spaces), with isotopic samples further differentiated by land use.Each soil sample was collected in-suite using a standardized auger method to a depth of 10 cm, minimizing contamination and ensuring consistency across samples^36^.

Soil samples with particle sizes less than 2 mm were used for analysis. After collection, the soils were air-dried at room temperature and then sieved through a 2 mm mesh to select only particles of this size for analysis^37,38^. To address potential inhomogeneity, several steps were taken to ensure consistency across samples. First, after sieving, the soil was thoroughly homogenized to guarantee uniform distribution of particles, minimizing the risk of bias in contaminant concentrations in the final sample^39,40^. Furthermore, to reduce the possibility of heterogeneity, a sub-sampling technique was employed, where multiple aliquots of the homogenized soil were taken for analysis. This ensured that the analytical results reflected the true composition of the soil, without being influenced by localized variations in contaminant distribution^41^. Additionally, to confirm the homogeneity of the prepared samples, duplicate samples were analyzed for selected parameters. The results of these tests demonstrated good agreement, effectively validating the accuracy and consistency of the sample preparation process. These measures were implemented to ensure that the data obtained accurately represents the true composition of the soils, without the influence of sampling or preparation errors^42^.

### Soil analysis for potentially toxic elements (PTEs)

Potentially Toxic Elements (PTEs) including Zn, Cu, Ni, Cd, Cr, As, and Pb in 53 surface soil samples from Sanandaj, Iran, were quantified using a Thermo Scientific iCAP RQ ICP-MS for its high sensitivity. Soil samples, sieved to < 2 mm, were digested (0.5 g subsample, 3:1 HNO₃:HCl aqua regia) in a Milestone UltraWAVE system, heated to 180 °C for 15 min, held for 10 min, diluted to 50 mL with ultrapure water, and filtered (0.45 μm PTFE)^43,44^. The ICP-MS was calibrated daily (TraceCERT standards, R² > 0.999), using Rh and In (10 µg/L) as internal standards^43^. Standard mode was used for Zn, Cu, Ni, Cd, and Pb, and KED mode with helium (4.5 mL/min) for As and Cr. Parameters included 1550 W plasma power, 0.9 L/min nebulizer flow, and 0.8 L/min auxiliary flow. limits of detection.

(LODs) and limits of quantification (LOQs), converted to mg/kg for soil, and precision metrics are in Supplementary Table S1. QA/QC involved NIST 2710a and NIST 8704 CRMs, procedural blanks (10% of samples), duplicate analyses, and calibration checks (10 µg/L standard). Matrix effects were minimized using internal standards and KED mode, but soil-type-specific evaluations were not conducted^43,44^.

### Advanced lead isotope analysis and environmental implications

An advanced methodology for lead (Pb) isotopic analysis was conducted using high-resolution multi-collector ICP-MS (Thermo-Finnegan Neptune) at Actlabs in Canada, achieving precise isotopic ratio measurements. Following the^45^ approach, measurement precision was ± 0.06% for ²⁰⁶Pb/²⁰⁴Pb, ± 0.05% for ²⁰⁷Pb/²⁰⁴Pb, ± 0.08% for ²⁰⁸Pb/²⁰⁴Pb, and ± 0.04% for ²⁰⁸Pb/²⁰⁶Pb. Certified Reference Material SRM 981 ensured calibration accuracy, enabling reliable differentiation of isotopic compositions to trace lead contamination sources in soils. This study integrated cutting-edge technology, international expertise, and robust QA/QC methodologies to deliver a reproducible dataset on metal concentrations and distributions. The isotopic analysis provides critical insights into anthropogenic and natural sources of lead, with significant implications for environmental management and policy formulation. By offering a detailed understanding of contamination pathways, the findings support sustainable practices and soil quality preservation, establishing a robust foundation for informed ecological stewardship and decision-making.

### PAHs analysis

A comprehensive analysis of fifteen Polycyclic Aromatic Hydrocarbon (PAH) compounds, identified as priority pollutants by the United States Environmental Protection Agency (USEPA), was conducted to assess environmental contamination in Sanandaj’s urban soils. The sample preparation for gas chromatography-mass spectrometry (GC-MS) involved extracting 10 g of dry weight from each of the 53 soil samples using a Soxhlet extractor, ensuring thorough and representative sampling. To enhance analytical precision, 2 g of activated copper were added to 200 milliliters of dichloromethane in the Soxhlet apparatus to facilitate sulfur removal and improve extraction efficiency [50]. Extraction was performed at 53 °C for 6 to 9 h to capture a comprehensive spectrum of PAH compounds. Post-extraction, samples were concentrated using a rotary evaporator and further purified via a silica gel column, with aliphatic hydrocarbons separated using n-hexane in the first stage, followed by PAH elution with a 70-milliliter mixture of dichloromethane and n-hexane (40:60, v/v) in the second stage. The final extract was reduced to 1 milliliter under a gentle nitrogen stream to optimize detection sensitivity for GC-MS analysis.

To verify PAH recoveries and ensure analytical accuracy, deuterated surrogate standards (naphthalene-d8, acenaphthene-d10, phenanthrene-d10, chrysene-d12, and perylene-d12) were spiked into each sample prior to extraction at a concentration of 50 µg/kg, following established protocols^46^. These surrogates, selected for their chemical similarity to target PAHs, were used to monitor extraction efficiency and correct for losses during sample preparation. Additionally, internal standards (fluoranthene-d10 and pyrene-d10) were added to the final extracts at 20 µg/kg before GC-MS analysis to account for instrumental variability and matrix effects. Recovery rates for the surrogate standards ranged from 85% to 105%, with relative standard deviations (RSD) below 10%, indicating high extraction efficiency and analytical reliability (see Supplementary Information, Table S6). Procedural blanks were analyzed with each batch to confirm the absence of contamination, and calibration curves were established using multi-point calibration standards (10–200 µg/L) with a correlation coefficient (R²) > 0.99. The GC-MS analysis was performed using an Agilent 7890B GC coupled with a 5977 A MS, following the methodology of^47^, ensuring precise identification and quantification of PAH compounds. This rigorous approach, incorporating surrogate and internal standards, provides robust and reliable data on PAH concentrations, supporting environmental monitoring and risk assessment in Sanandaj’s urban soils.

### Human health risk assessment

The evaluation of health risks linked to Potentially Toxic Elements (PTEs), within this study adopts a detailed, multi-tiered methodology grounded in the protocols set forth by the United States Environmental Protection Agency^48^.

In this method, the hazard quotient (HQ) and cancer risk (CR) were applied to determine non-carcinogenic and carcinogenic risks of individual toxic metals, respectively^44^.

Humans are exposed to dust and surface soil throughout three main exposure including ingestion (D_ing_), inhalation (D_inh_), and dermal absorption (D_dermal_)^49^. These factors can be calculated based on the exposure factors handbook^48,50^. The average daily dose (ADD) taken by every exposure pathway is evaluated by Eqs. (1)–(3) for daily intake^48,50^.

Based on the USEPA (1997), IngR and InhR are the ingestion and inhalation rates. Ingestion exposure rate of soil and dust is 200 m^3^/day and 100 m^3^/day for adults, respectively. Inhalation exposure rate for children and adults is 7.6 m^3^/day and 20 m^3^/day, respectively^50,51^. C is the mean value (mg/kg) of each toxic metal. EF and ED are exposure frequency and exposure duration, respectively. The EF value is 350 day/year for children and adults^48^; ED value is 30 years for adults and 6 years for children (EPA 2001); CF is the conversion factor (1 × 10^−6^ kg/mg) for both children and adults; SA is the surface area of the skin which is in contact with dust; SA value is 2800 cm^2^ and 3300 cm^2^ for children and adults, respectively^48^. AF is the skin adherence factor, and AF factor is 0.2 mg/cm^2^/h for children and 0.7 mg/cm^2^/h for adults^48^. ABF is the dermal absorption factor (0.001).

Particle emission factor (PEF) is 1.36 9 10 9 m^3^/kg (EPA 2001). BW factor is the mean of body weight for children (15 kg) and adults (70 kg), and AT is the averaging time (for non-carcinogens, AT = 365× ED days; for carcinogens, AT = 70 × 365 = 25,550 days).

1 $$\:{ADD}_{ing}=\frac{C\times\:IngR\times\:CF\times\:EF\times\:ED}{BW\times\:AT}$$

2 $$\:{ADD}_{inh}=\frac{C\times\:IngR\times\:CF\times\:EF\times\:ED}{PEF\times\:BW\times\:AT}$$

3 $$\:{ADD}_{dermal}=\frac{C\times\:SA\times\:CF\times\:AF\times\:ABF\times\:EF\times\:ED}{BW\times\:AT}$$

According to non-cancer risk of individual toxic metals and a specific reference dose (RfD), the hazard quotient (HQ) was calculated (Man et al. 2010). Specific reference dose (mg/kg day) was an estimation of the highest acceptable risk to a population through a life. The hazard index (HI) is the addition of HQs and was applied to assess three main exposure pathways individually (Eqs. 4–5). HI values < 1 indicate that there are no obvious adverse health effects of toxic metals for residential population, whereas, if HI > 1 exceeds one, it indicates that hostile health effects can occur^49^.

4 $$\:{HQ}_{i}=\sum\:\frac{{ADD}_{i}}{{R}_{i}{D}_{i}}$$

5 $$\:HI=\sum\:{HQ}_{i}$$

In general, carcinogenic risk is considered as possibility of cancer during life because of contact to carcinogenic hazards^52^. The following equation (Eq. 6) was used to calculate carcinogenic risks of toxic metals during a lifetime^49^:

6 $$\:CR\left(\mathrm{i}\right)=\left({\mathrm{A}\mathrm{D}\mathrm{D}}_{\mathrm{i}\mathrm{n}\mathrm{g}\mathrm{e}\mathrm{s}\mathrm{t}\mathrm{i}\mathrm{o}\mathrm{n}\left(\mathrm{i}\right)}+\:{\mathrm{A}\mathrm{D}\mathrm{D}}_{\mathrm{i}\mathrm{n}\mathrm{h}\mathrm{a}\mathrm{l}\mathrm{a}\mathrm{t}\mathrm{i}\mathrm{o}\mathrm{n}\left(\mathrm{i}\right)}+\:{\mathrm{A}\mathrm{D}\mathrm{D}}_{\mathrm{d}\mathrm{e}\mathrm{r}\mathrm{m}\mathrm{a}\mathrm{l}\left(\mathrm{i}\right)}\right)\times\:\mathrm{S}\mathrm{F}\mathrm{i}\left(\mathrm{i}\right)$$

Carcinogenic slope factor was defined as (SF) (per mg/kg/day), which transfers the assessed daily intake of a contaminant during lifetime of exposure to the incre-mental risk of an individual developing cancer^53^. Risks higher than 1 × 10^−4^ are considered as unacceptable, risks values < 1 × 10^−6^ are not regarded as significant, and values between 1 × 10^−4^ and 1 × 10^−6^ are considered as a standard range^22^.

### Potential ecological risk

The Potential Ecological Risk Index (PERI), developed by Hakanson^54^ has been applied to evaluate ecological risks from toxic metals in urban surface soils. It measures metal concentrations in soil, assigns toxicity factors based on environmental harm, calculates individual metal risks, and aggregates them into a single Risk Index (RI) to assess overall ecological risk and guide environmental management^44^. It measures metal concentrations in media such as soil or water, assigns toxicity factors based on environmental harm, calculates individual metal risks, and aggregates them into a single Risk Index (RI) to assess overall ecological risk and guide environmental management (Eq. 7).

7 $$\:\mathrm{R}\mathrm{I}=\sum\:{E}_{r}^{i}=\sum\:{T}_{r}^{i}(\:{C}_{s}^{i}/{C}_{n}^{i})$$

Where:

$\:{E}_{r}^{i}$ : The potential ecological risk index of a single element.

$\:{T}_{r}^{i}$ : The toxic response factor for each of the PTEs.

$\:{C}_{s}^{i}$ : The concentrations of toxic metals in the dust samples.

$\:{C}_{n}^{i}$ : The reference values of PTEs.

The toxic-response factors for Pb, Zn, Cu, Cr, Cd, and As are 5, 1, 5, 2, 30, and 10, respectively^55^. Table 1 shows the grading standards of the potential ecological risk of toxic metals.

In ecological risk assessment, toxic-response factors reflect the relative toxicity of metals, assigning weights to evaluate their environmental impact. The toxic-response factors for key metals are: Pb (5), Zn (1), Cu (5), Cr (2), Cd (30), and As (10). These values indicate the varying ecological harm of each metal, with Cd posing the highest risk. These factors are used in models like PERI to assess and manage ecological risks.

Toxic-response factors highlight the ecological harm of metals, with Cd’s high factor (30) indicating significant risk compared to metals with lower factors. Table 1 categorizes ecological risks into levels (e.g., low, moderate, high) based on metal concentrations and toxicity factors. These standards, detailed in the study’s documentation, guide environmental management and mitigation decisions.

**Table 1:** Assessment metrics and classification levels for potential ecological Risk.

Er	Status of ecological risk for a single metal	RI	Grade of potential ecological risk to the environment
40 < Er	Low	150 < RI	Low risk
80 < Er ≤ 40	Moderate	300 < RI ≤ 150	Moderate risk
160 < Er ≤ 80	Considerable	600 < RI ≤ 300	Considerable risk
320 < Er ≤ 160	High	600 ≥ RI	High risk
320 ≥ Er	Very high		

### Ecological risk of PAHs

In this research, a combination of environmental indicators and health risk indices was employed to evaluate Polycyclic Aromatic Hydrocarbons (PAHs) compounds^22,56,57^. To assess the potential risks and health impacts of PAH compounds in soil and water environments, the study utilized the following indices: the Soil Screening Quick Reference Tables (SQTs) developed by Li, et al.^1^ for evaluating the risk of PAH contamination in soil, the Standardized Oil and Grease Survey (SOGS) for determining PAH concentrations in soil, and the Effects Range Median (ERM) along with Effects Range Low (ERL) derived from analyzing the 10th and 50th percentiles of relevant biological effect data caused by PAH exposure. The ERL represents the threshold concentration below which minimal adverse effects are expected, while the ERM indicates the concentration above which potential adverse effects may occur. Additionally, concentrations that exceed the ERL but remain below the ERM are associated with possible effects, whereas concentrations surpassing the ERM are linked to probable effects.

### Evaluation of carcinogenic risk of PAHs

Detecting Polycyclic Aromatic Hydrocarbons (PAHs) in Sanandaj’s urban surface soils is concerning due to their toxicological risks, especially in industrial and traffic-heavy areas. Quantifying this toxicity is crucial for environmental impact assessment. Toxicity Equivalency Factors (TEFs), also termed Toxicity Equivalence Potencies (TEPs), are pivotal in evaluating PAH toxicity by assigning values relative to benzo[a]pyrene (BaP), expressed as BaP equivalents (BaPeq). The BaPeq model, introduced by^58^, quantifies the toxic potency of PAHs, forming the basis for calculating Toxic Equivalent Concentration (TEQ). Studies by^59^ and^60^ provide refined TEFs, enabling precise TEQ assessments for PAHs in urban soils.

The BaPeq model, built on^61^ and extended by^62^, serves as a benchmark for gauging PAH harm. BaP, classified as a Group 1 carcinogen by the IARC, is a standard for assessing PAH carcinogenicity. TEFs from^63^ quantify the relative toxicity of PAHs compared to BaP. The Total Equivalent Concentration (TEQ), calculated by aggregating PAH contributions adjusted by TEFs, uses BaP as a surrogate to estimate carcinogenic potential. This approach enhances the accuracy of environmental risk assessments and supports informed regulatory decisions for managing PAH contamination in urban ecosystems.

The toxic equivalent concentration (BaPeq) for each individual PAH compound can be calculated using the following Eq. (8):

8 $$\:{BaPeq}_{i}={PAH}_{i}\times\:{TEF}_{i}$$

The Total Equivalent Concentration (TEQ) is then determined by summing the contributions from all PAHs, adjusted by their respective Toxic Equivalency Factors, as shown in Eq. (9):

9 $$\:TEQ=\sum\:_{i}^{n}\left({PAH}_{i}\times\:{TEF}_{i}\right)$$

PAHi stands for Polycyclic Aromatic Hydrocarbons, and TEF represents the Toxicity Equivalency Factor, a mathematical model used to assess the toxic effects of PAHs. Additionally, TEQ stands for Toxicity Equivalents, another calculation used to estimate the overall toxicity of a mixture containing PAHs. Numerous studies have evaluated the potential health risks associated with exposure to Polycyclic Aromatic Hydrocarbons (PAHs) and their effects on Incremental Lifetime Cancer Risk (ILCR) following the guidelines established by the Environmental Protection Agency (EPA)^3,64^. The ILCR is determined through mathematical models incorporating equations for the three primary exposure routes: ingestion, dermal contact, and inhalation.

The Incremental Lifetime Cancer Risk (ILCR) for the three primary exposure routes—ingestion, dermal contact, and inhalation can be calculated using the Eqs. (10–12), respectively.

10 $$\:{LCRs}_{Ingestion}=\frac{cs\times\:\:\left({CSF}_{Ingestion}\times\:\sqrt[3]{\left(\frac{BW}{70}\right)}\right)\:\times\:{IR}_{Ingestion}\times\:EF\times\:ED}{BW\times\:AT\times\:{10}^{6}}$$

11 $$\:{LCRs}_{Dermal}=\frac{cs\times\:\:\left({CSF}_{Dermal}\times\:\sqrt[3]{\left(\frac{BW}{70}\right)}\right)\:\times\:SA\times\:AF\times\:ABS\times\:EF\times\:ED}{BW\times\:AT\times\:{10}^{6}}$$

12 $$\:{LCRs}_{Inhalation}=\frac{cs\times\:\:\left({CSF}_{Inhalation}\times\:\sqrt[3]{\left(\frac{BW}{70}\right)}\right)\:\times\:{IR}_{Inhalation}\times\:EF\times\:ED\:}{BW\times\:AT\times\:PEF}$$

Polycyclic aromatic hydrocarbon (PAH) concentrations in samples were assessed by measuring benzo[a]pyrene (BaP) levels and applying Toxicity Equivalency Factors (TEFs) as established by^61^. The Cancer Risk Factor (CSF) quantifies the average daily cancer risk, particularly lung cancer, from BaP exposure, measured in micrograms per cubic meter per day. Parameters such as body weight (BW), exposure duration (AT), exposure frequency (EF), lifetime exposure duration (ED), inhalation rate (IR Inhalation), ingestion rate (IR Ingestion), exposed skin surface area (SA), absorption fraction (AF), absorption factor (ABS), and particle emission factor (PEF) were used for precise PAH assessment (Table 2). Cancer risk factors for BaP exposure via ingestion (CSF Ingestion: 3.7), dermal contact (CSF Dermal: 25), and inhalation (CSF Inhalation: 85.3) micrograms per kilogram per day were estimated for children (1–6 years) and adults (7–31 years) using the^65^. The TEF methodology evaluated BaP’s carcinogenic potential across contaminated soil, air, and dust through inhalation, ingestion, and dermal pathways, yielding cancer risk estimates of 6–10 cases per million individuals exposed^4,39^.

**Table 2:** Parameters used in cancer risk assessment (ILCR)^66^.

Factor	Unit	Children	Adults
BW	kilogram	15	61.5
EF	Day per year	180	180
ED	year	6	24
IRInh	cubic meters per day	10	20
IRIng	cubic meters per day	200	100
SA	square centimeter per day	2800	5700
AF	milligram per square centimeter	0.2	0.07
ABS	-	0.13	0.13
AT	day	365 × 70	365 × 70
PEF	cubic meter per kilogram	1.36 × 109	1.36 × 109

### Potential ecosystem risk

To evaluate the potential hazards associated with specific PAHs in the city of Sanandaj, the concentrations of identified PAH species in surface soil were compared to their corresponding benchmark values obtained in this study, utilizing the Risk Quotient (RQ) approach to assess the possible ecological risks of PAHs in surface soil Yan, et al.^67^ with RQ values determined by the following equation (Eq. 13).

13 $$\:RQ=\frac{{C}_{PAHS}}{{C}_{QV}}$$

The Risk Quotient (RQ) indicates the potential ecological hazards posed by PAHs, where higher RQ values reflect increased risk levels.

In this study, CPAHs refer to the measured concentrations of specific PAH compounds in the soil samples, and CQV denotes the corresponding average qualitative benchmark values for these PAHs. The qualitative standards employed were Negligible Concentration (NCs) and Maximum Permissible Concentration (MPCs) under typical conditions, as outlined by Yan, et al.^67^. Consequently, the RQ values for NCs (RQ NCs) and MPC (RQ MPC) were calculated using Eqs. (14) and (15), respectively.

14 $$\:R{Q}_{NCs}=\frac{{C}_{PAHS}}{{C}_{Qv\left(NCS\right)}}$$

15 $$\:R{Q}_{MPCS}=\frac{{C}_{PAHS}}{{C}_{Qv\left(MPCS\right)}}$$

CPAHs denote the concentration of specific PAHs in the surface soil. The Risk Quotients for Negligible Concentrations (RQ_NCs) and Maximum Permissible Concentrations (RQ_MPCs) represent the qualitative thresholds for PAHs present in the soil. These Risk Quotients are applied exclusively to eight particular PAH compounds during the ecosystem risk assessment. For the remaining five PAHs-Acenaphthylene (Acy), Fluorene (Fl), Pyrene (Pyr), Benzo(b)fluoranthene (B(b)F), and Dibenz(a, h)anthracene (DBA)-the assessment utilizes Toxic Equivalency Factors (TEFs) as described by Nisbet and Lagoy^61^. Specifically, Acy, Fl, and Pyr, which have TEFs of 0.001, similar to Anthracene (Ant), are evaluated using these low TEF values. In contrast, B(b)F, with a TEF of 0.1 akin to Benzo(a)anthracene (BaA), is assessed using a higher TEF. DBA, sharing a TEF of 1 with Benzo(a)pyrene (BaP), is evaluated accordingly. The cumulative Risk Quotients for all PAHs, denoted as RQ_ΣPAHs (NCs) and RQ_ΣPAHs (MPCs), are calculated using the equations presented by Cao, et al.^56^.

The cumulative Risk Quotients for all PAHs (RQ_ΣPAHs) for different concentration categories—Negligible Concentrations (NCs) and Maximum Permissible Concentrations (MPCs) are calculated using the Eqs. (16–18).

16 $$\:{RQ}_{\sum\:PAHs}=\:\sum\:_{i=1}^{15}{RQ}_{i}({RQ}_{i}\ge\:1)$$

17 $$\:{RQ}_{\sum\:{PAHs}_{\left(NCs\right)}}=\:\sum\:_{i=1}^{15}{RQ}_{\left(NCs\right)}\left({RQ}_{\left(NCs\right)}\ge\:1\right)$$

18 $$\:{RQ}_{\sum\:{PAHs}_{\left(MPCs\right)}}=\:\sum\:_{i=1}^{15}{RQ}_{\left(MPCs\right)}({RQ}_{\left(MPCs\right)}\ge\:1)$$

To evaluate the potential ecological risks associated with polycyclic aromatic hydrocarbons (PAHs) in Sanandaj city, the concentrations of specific PAH species in surface soil were measured and compared against their respective benchmark values using the Risk Quotient (RQ) approach^68^. The RQ value indicates the potential risk posed by PAHs within the ecosystem, where higher RQ values denote greater risk levels. Specifically, CPAHs represent the concentration of individual PAHs in the surface soil. At the same time, RQ (NCs) and RQ (MPCs) correspond to the qualitative thresholds for Negligible Concentrations (NCs) and Maximum Permissible Concentrations (MPCs), respectively. These Risk Quotients were applied to eight specific PAH compounds during the ecosystem risk assessment. For the additional five PAHs-Acenaphthylene (Acy), Fluorene (Fl), Pyrene (Pyr), Benzo(b)fluoranthene (B(b)F), and Dibenz(a, h)anthracene (DBA)-Toxic Equivalency Factors (TEFs) were utilized as^61^. Specifically, Acy, Fl, and Pyr, which have TEFs of 0.001, similar to Anthracene (Ant), were assessed using these low TEF values. In contrast, B(b)F, with a TEF of 0.1, comparable to Benzo(a)anthracene (BaA), was evaluated using a higher TEF. At the same time, DBA, sharing a TEF of 1 with Benzo(a)pyrene (BaP), was assessed accordingly. The cumulative Risk Quotients for all PAHs, denoted as RQ∑PAHs (NCs) and RQ∑PAHs (MPCs), were calculated based on the equations outlined by^39^, Cao, et al.^56^. Essentially, an RQ (NCs) value indicates that the PAHs present are likely of minimal concern and may be disregarded. In contrast, an RQ (MPCs) value suggests that PAH contamination is significant enough to require prompt control measures and remedial actions. In scenarios where RQ (NCs) exceeds 1 while RQ (MPCs) remains below 1, the PAH pollution is considered to be of moderate concern, necessitating the implementation of specific control strategies or remediation efforts (see Table 3).

**Table 3:** Risk classification of individual PAHs and ΣPAHs.

Individual PAHs			Σ ΠΑΗ_Σ_		
	RQ_(NCS)_	RQ_(MPCS)_		RQ _SPAHS (NCS)_	RQ _SPAHS(MPCS)_
Risk-free	< 1	< 1	Risk-free	< 1	< 1
			Low-risk	≥ 1, < 800	< 1
Moderate-risk	≥ 1	< 1	Moderate-risk1	≥ 800	< 1
			Moderate-risk2	≤ 800	≥ 1
High-risk	≥ 1	≥ 1	High-risk	≥ 800	≥ 1

### Isotopic ratios

Lead (Pb) exists in nature as four stable isotopes: ^204^Pb, ^206^Pb, ^207^Pb, and ^208^Pb. Of these, three isotopes ^206^Pb, ^207^Pb, and ^208^Pb are produced through the radioactive decay of uranium-238 (^238^U), uranium-235 (^235^U), and thorium-232 (^232^Th), respectively. These lead isotopes’ formation pathways and sources result in significant variations in their relative abundances^69,70^. Different origins, including anthropogenic activities and geological deposits, exhibit unique isotopic signatures and ratios^40,70^.

Lead isotopic ratios remain unchanged during industrial processes and environmental transformations, preserving the original ratios present in primary lead ores^71,72^. This stability makes lead isotopic ratios valuable for tracing lead contamination sources and movement pathways in environmental studies^70,73–75^.

Identifying lead pollution sources in soils through isotopic analysis relies on distinguishing between the isotopic compositions of natural materials-such as unpolluted soils and bedrock—and those of anthropogenic lead contaminants derived from lead ores^76^. Lead ores, predominantly galena, typically display elevated Pb/Th and Pb/U ratios compared to common rocks, and their isotopic ratios remain stable over time since their formation^77^. Consequently, lead ores possess a distinct and consistent isotopic signature, often characterized by low ratios of non-radiogenic isotopes like ²⁰⁴Pb/²⁰⁶Pb^77,78^.

Conversely, lead found in unpolluted soils originates from the weathering of bedrock, which is influenced by the isotopic ratios of uranium to lead (U/Pb) and thorium to lead (Th/Pb) in the source materials. These ratios evolve, resulting in soil lead isotopic profiles that typically reflect the radiogenic ²⁰⁴Pb/²⁰⁶Pb ratios associated with natural geological processes^70,76^. This distinction allows for the differentiation between natural and anthropogenic lead sources in environmental assessments.

## Results

### Concentration of PTEs

The summary of the statistical status of PTEs concentrations and physical properties of urban soil samples in Sanandaj is presented in Table 4. The average Pb concentration in the studied area is 125.55 mg/kg, ranging from 74 to 270 mg/kg. The Cu concentration in the studied samples ranges from 34 to 240 mg/kg, with an average of 56.83 mg/kg.

The average concentration of Ni in the samples is 56.19 mg/kg. The Pb, Cu, and Ni concentrations in all studied samples are significantly higher than those in the upper continental crust (UCC). The lead concentration ranges from 5 to 128 mg/kg, with an average of 27.04 mg/kg. In 75% of the studied samples, the Pb concentration exceeds that of the upper continental crust, averaging 111.47 mg/kg. The average concentration of Cr varies between 72 and 138 mg/kg, while Cd ranges from 0.1 to 6.24 mg/kg. All analyzed samples show Cd and Cd levels that exceed those typically in the upper continental crust.

The concentration of As in the samples varies from 6.2 to 38 mg/kg, with an average of 4.62 mg/kg. Compared to the upper continental crust, As concentrations in 22% of the samples exceed those in the upper continental crust. All analyzed samples exhibit Cr and Cd concentrations that surpass those typically in the upper continental crust. Additionally, the average metal concentrations in the studied samples follow a decreasing trend as Zn > Cr > Ni > Cu > Pb > As > Cd.

**Table 4:** Descriptive statistics of PTEs concentrations and physical properties of surface soil samples in Sanandaj City.

PTEs	Min	Max	mean	STDEV	Cv	Kurtosis	Skewness
Zn	74	270	125.55	40.77	0.32	1.93	4.46
Cu	34	240	56.83	29.84	0.52	4.7	26.96
Ni	37	78	56.19	9.17	0.16	0.14	− 0.32
Pb	5	128	27.04	20.11	0.74	2.66	10.94
Cr	72	138	111.47	16.28	0.15	− 0.56	−0.49
Cd	0.19	1.6	0.24	0.19	0.77	7.24	52.56
As	2.6	38	4.62	4.81	1.04	6.48	44.94
pH	8.09	8.83	8.52	0.16	0.02	−0.51	− 0.27
EC	223	3160	673.68	557.79	0.83	3.32	12.13

### Comparison of PTE concentrations in surface soil of Sanandaj City with other urban areas worldwide

Assessing the environmental status of potentially toxic elements (PTEs) in urban soils is crucial for understanding ecological and public health risks. In the absence of standardized guidelines for soil sampling and geochemical analysis in urban environments, particularly within Iran, a comparative study of average PTE concentrations in the surface soil of Sanandaj city against various global urban areas offers valuable insights. Table 5 presents the average concentrations of PTEs (in mg/kg) in the surface soils of Sanandaj and selected cities worldwide, facilitating a comprehensive evaluation of pollution levels and potential sources.

Sanandaj exhibits elevated concentrations of (Zn, 125.55 mg/kg), (Cr, 111.47 mg/kg), and (Ni, 56.19 mg/kg) compared to the Upper Continental Crust (UCC) values, which are 31 mg/kg for Zn, 35 mg/kg for Cr, and 20 mg/kg for Ni. These elevated levels in Sanandaj can be attributed to its industrial landscape, including metal processing and manufacturing sectors that release Zn, Cr, and Ni through galvanization, stainless steel production, and alloy manufacturing^79^. Additionally, vehicular traffic contributes to Zn and (Cu) accumulation via tire wear and brake degradation. The high Zn concentration suggests extensive use of galvanized materials and possible industrial effluent discharge, while elevated Cr and Ni levels indicate the region’s prevalent stainless-steel production and alloy manufacturing activities^79^.

In comparison, Arak presents extraordinarily high Cu (2839.77 mg/kg) and (Pb, 979.4 mg/kg) concentrations, surpassing those in Sanandaj (Cu: 56.83 mg/kg; Pb: 27.04 mg/kg). Arak is a major industrial hub in Iran, known for its petrochemical and steel industries, which significantly contribute to Cu and Pb pollution. The intensive copper-related industrial activities, including refining and processing facilities, emit substantial amounts of copper into the environment, resulting in the exceptionally high soil concentrations observed. Similarly, Tehran, the capital city, has a diverse industrial base encompassing manufacturing, energy production, and extensive vehicular traffic, leading to elevated Pb (257.4 mg/kg) and Cu (225.3 mg/kg) levels compared to Sanandaj. This reflects Tehran’s status as a significant metropolitan hub with a broad spectrum of pollution sources.

Ottawa, Canada, exhibits lower concentrations of Cu (38 mg/kg), Ni (15 mg/kg), Cr (42 mg/kg), and arsenic (As, 1 mg/kg) compared to Sanandaj, likely due to stringent environmental regulations and effective emission control technologies that mitigate PTEs emissions from both industrial and vehicular sources. These regulatory measures and advanced pollution control technologies result in significantly lower PTE concentrations in Ottawa’s surface soils. Conversely, Isfahan shows higher Zn (470.36 mg/kg) and As (16.17 mg/kg) levels than Sanandaj, attributable to its diverse industrial base, including petrochemicals and metal processing industries, which are major sources of these metals. The extensive use of petrochemical products and metal alloys in Isfahan contributes to the elevated Zn and As levels through industrial emissions and effluent discharges.

Shiraz and Naples, Italy, present lower concentrations of several PTEs than Sanandaj, which can be linked to their different industrial activities and more effective environmental management practices. Shiraz, with its diversified economy and focus on sectors with lower PTEs emissions, shows lower Zn (56.91 mg/kg) and Cr (366.16 mg/kg) levels. Naples benefits from effective pollution control measures and a different industrial focus, resulting in lower levels of Ni (11.6 mg/kg) and Cr (15.3 mg/kg) compared to Sanandaj, which may indicate a reduced emphasis on nickel-intensive industries and better-managed chromium emissions.

With its substantial metallurgy and manufacturing sectors, Xiangyang, China, has higher Pb (80.92 mg/kg) and As (27.13 mg/kg) levels than Sanandaj. In contrast, Ahvaz, dominated by oil refining and petrochemicals, shows higher Zn (288 mg/kg) and Cu (113 mg/kg) levels. These elevated concentrations in Xiangyang and Ahvaz reflect their intensive industrial activities, which release significant amounts of lead and arsenic from metallurgical processes and Zn and Cu from petrochemical operations. In contrast, Sanandaj maintains higher levels of Zn and Ni, reflecting localized industrial practices that differ from those in Xiangyang and Ahvaz, possibly due to variations in the types of industries and the effectiveness of pollution control measures.

Andhra Pradesh, India, exhibits lower Zn (97.3 mg/kg) and Pb (13.3 mg/kg) concentrations than Sanandaj, likely due to its predominantly agricultural practices and less intensive metal processing industries. The lower PTEs deposition in Andhra Pradesh’s soils can be attributed to reduced industrial emissions and the predominance of farming activities that do not contribute significantly to Zn and Pb contamination. This contrasts with Sanandaj’s more industrially driven PTE profile, highlighting the impact of industrial intensity on soil contamination levels.

Globally, Sanandaj’s soil presents higher Zn, Cu, Ni, and Cr concentrations than world soil averages (Zn: 70 mg/kg; Cu: 38.9 mg/kg; Ni: 24 mg/kg; Cr: 67 mg/kg), while Pb (27.04 mg/kg), cadmium (Cd, 0.24 mg/kg), and As (4.62 mg/kg) levels are comparable or lower. This indicates that certain metals are of significant concern in Sanandaj due to localized industrial emissions, vehicular traffic, and possibly geological factors. In contrast, others are managed more effectively or are less prevalent due to differing local industrial practices and regulatory measures^80^. The elevated levels of Zn and Ni, in particular, suggest specific industrial sources and highlight the need for targeted environmental controls^80^.

This comprehensive analysis highlights the specific PTE concentration levels in Sanandaj compared to other urban areas and elucidates the underlying sources and contributing factors. Understanding these dynamics is essential for developing effective environmental management strategies tailored to the unique contamination profile of Sanandaj, ensuring the protection of both ecosystem health and human well-being.

**Table 5:** The average concentration of PTEs (mg/kg) in the surface soil of Sanandaj and other cities of the world.

City	Zn	Cu	Ni	Pb	Cr	Cd	As	References
Sanandaj	125.55	56.83	56.19	27.04	111.47	0.24	4.62	This Study
(UCC)*	31	29	20	15	35	0.09	4.8	^81^
Arak	109.3	2839.77	729.87	979.4	733.67	7.68	9.5	^82^
Ortawa/Canada	101	38	15	33	42	0.3	1	^83^
Isfahan	470.36	92.75	61.65	179.95	80.57	2.17	16.17	^84^
Shiraz	56.91	53.66	106.16	17.83	366.16	0.25	4.23	^85^
Napoli/Italy	223	94	11.6	204	15.3	0.58	13.4	^86^
Tehran	873.2	225.3	34.8	257.4	33.5	10.7	-	^87^
Ahvaz	288	113	57	59	50	0.52	11.3	^22^
Andhra Pradesh/India	97.3	74	50.1	13.3	126	-	6.1	^88^
World Soil	70	38.9	24	27	67	0.41	6.83	^89^
World Background	90	30	50	35	70	0.35	6	^90^

* Upper Continental Crust.

### Potential ecological risk assessment of PTEs in Sanandaj City

In this study, the Potential Ecological Risk (Er) of various potentially toxic elements (PTEs) in the surface soil of Sanandaj city was evaluated using the Hakanson method^54,91^. The results, summarized in Table 6, reveal that the average Er values for the assessed PTEs rank in descending order as follows: Cd > Ni > Cu > As > Pb > Cr > Zn. Specifically, the mean Er values for Cu, Pb, As, Ni, Cr, and Zn are all below 40, indicating a low ecological potential risk for these metals. In contrast, Cd exhibits a significantly higher Er value, underscoring its substantial ecological risk compared to the other toxic elements examined.

The Potential Ecological Risk Index (RI) was further calculated to assess the cumulative risk of multiple metals in the surface soil. The RI serves as an overarching indicator of environmental sensitivity to toxic metal contamination, providing a general assessment of pollution severity^44^. According to the RI values presented in Tables 6 and 99.45% of the soil samples are categorized as low risk, 5.7% as moderate risk, and 1.9% as high risk. These findings suggest that while most areas within Sanandaj exhibit minimal ecological risk from PTEs, there are localized zones where contamination levels necessitate attention and potential remediation measures^79,80^.

The elevated Er value for Cd can be attributed to several factors related to industrial and anthropogenic activities in Sanandaj. Critical sources of Cd contamination include industrial emissions from metal plating, battery manufacturing, and non-ferrous metal processing, which release Cd into the environment through atmospheric deposition and effluent discharge^92^. Additionally, vehicular traffic contributes to Cd accumulation via brake wear and tire degradation, with high traffic density and older vehicles exacerbating this issue^80^. Improper disposal and management of industrial and municipal waste can also lead to Cd leaching into the soil, with landfills and waste treatment facilities without adequate safeguards serving as significant sources of contamination^92^.

The low Er values reflect controlled emission sources and effective environmental management practices for the other metals. Zn levels are elevated due to galvanization processes and industrial effluents but remain below critical risk levels through regulatory measures. Cu, released from industrial activities such as metal processing and vehicular emissions, is kept within safe ecological thresholds. Nickel and chromium, primarily from stainless steel production and alloy manufacturing, have concentrations managed by emission controls. As and Pb, originating from industrial emissions and historical use of leaded materials, have their levels mitigated through effective pollution control technologies^44,93^.

Several patterns emerge when comparing the ecological risk in Sanandaj with other urban centers. Cities like Arak and Tehran exhibit higher concentrations of Cu and Pb due to their intensive petrochemical and steel industries, indicating more severe ecological risks than Sanandaj. Ottawa, Canada, demonstrates lower Er values for Cu, Pb, As, Ni, Cr, and Zn, attributed to stringent environmental regulations and advanced emission control technologies, resulting in minimal ecological risk from these metals. With higher Zn and As levels due to its diverse industrial base, Isfahan highlights differences in pollution sources and management. In contrast, Shiraz and Naples, Italy, present lower concentrations of several PTEs than Sanandaj, which can be linked to their different industrial activities and more effective environmental management practices (Table 5).

The environmental and health implications of elevated concentrations of Zn, Cr, and Ni in Sanandaj’s surface soil are significant. While Zn and Cu are essential trace elements, their excessive presence can disrupt soil microbial communities and inhibit plant growth. Chromium and nickel are recognized carcinogens capable of contaminating groundwater and entering the food chain, posing severe health hazards to the local population. Cadmium and arsenic, even at lower concentrations, are highly toxic and can accumulate in biological systems, leading to chronic health issues such as kidney damage and cancer^94^.

To address the elevated ecological risks associated with PTEs in Sanandaj, several mitigation strategies are recommended. Implementing enhanced monitoring through comprehensive soil tracking programs will help regularly assess PTE concentrations and identify pollution hotspots for timely intervention. Stricter industrial regulations and robust waste management practices in key sectors will minimize PTEs emissions. Additionally, promoting cleaner vehicle technologies and improving public transportation infrastructure will reduce vehicular emissions contributing to soil contamination. Employing soil remediation techniques such as phytoremediation, soil washing, and stabilization can decrease the bioavailability of PTEs and mitigate soil contamination. Increasing community awareness regarding the sources and risks of PTEs pollution will encourage environmentally friendly practices, and developing localized environmental policies tailored to Sanandaj’s specific PTE profile will ensure effective management and reduction of PTEs pollution.

In conclusion, the Potential Ecological Risk assessment reveals that Sanandaj city experiences elevated concentrations of specific PTEs, particularly Cd, Ni, Cu, As, Pb, Cr, and Zn, with cadmium posing a significant ecological risk. These elevated levels are primarily attributable to localized industrial activities, vehicular emissions, and possibly geological factors. Comparative analysis with other urban areas underscores the influence of distinct industrial profiles, regulatory frameworks, and environmental management practices on PTE concentrations. Addressing the elevated PTE levels in Sanandaj necessitates targeted environmental policies, effective pollution control measures, and ongoing monitoring to safeguard ecosystem integrity and public health. Compared with other urban areas, understanding Sanandaj’s unique contamination patterns is essential for developing effective strategies to mitigate the environmental and health impacts of PTEs pollution, ensuring a sustainable and healthy environment for its residents^79^.

**Table 6:** Distribution of potential ecological risk index (Er) of PTEs.

PTEs	Er				
	Min	Max	Mean	STDEV	Skewness
Zn	2.39	8.71	4.05	1.33	1.93
Cu	5.86	41.38	9.8	5.19	4.7
Ni	9.25	19.5	14.05	2.31	0.14
Pb	1.67	42.67	9.01	6.77	2.66
Cr	4.11	7.89	6.37	0.94	−0.56
Cd	63.33	533.33	91.32	63.41	7.24
As	5.42	79.17	9.62	10.12	6.48

### Human health risk

In assessing the human health risks associated with potentially toxic elements (PTEs) in the surface soil of Sanandaj city, a comprehensive evaluation was conducted focusing on both carcinogenic and non-carcinogenic risks for adults and children across three primary exposure pathways: ingestion, dermal contact, and inhalation. The results in Table 7 indicate that all studied Metals-Zn, Cu, Ni, Pb, Cr, Cd, and As-exhibit Hazard Quotients (HQ) below the threshold of 1 across all exposure pathways for both populations. According to the United States Environmental Protection Agency^95^, an HQ value below 1 suggests that non-carcinogenic adverse health effects are unlikely, while an HQ exceeding 1 would indicate potential health concerns. In this study, the Hazard Index (HI), which aggregates the HQs for a comprehensive risk assessment, also remains below 1 for all metals except for Cd and As in specific scenarios. This overall assessment suggests that non-carcinogenic risks from these metals are generally negligible for both adults and children in Sanandaj city.

However, it is noteworthy that children exhibit higher HQ values than adults, particularly in the ingestion and dermal contact pathways. This heightened vulnerability is attributed to children’s behaviors, such as hand-to-mouth activities, and their higher ingestion rates relative to body weight. The carcinogenic risk assessment reveals a more concerning scenario despite the low non-carcinogenic risks. The Cancer Risk (CR) values for five carcinogenic PTEs-Cr, Ni, Pb, cadmium Cd, and As-exceed the acceptable thresholds the Phillips and Moya^95^ set for adults and children. Specifically, chromium poses the highest carcinogenic risk, followed by arsenic, nickel, cadmium, and lead. The order of carcinogenic risk in children is Cr > As > Ni > Cd > Pb, while in adults, the trend is similar, although cadmium presents a higher risk than nickel. According to US EPA standards, a CR value exceeding 4 × 10^−6^ is considered unacceptable, and in this study, all assessed CR values surpass this limit, indicating a significant health threat. This elevated carcinogenic risk underscores the potential for increased cancer incidence within the exposed populations, particularly among children who are more susceptible to environmental contaminants.

Comparative analysis with other studies further contextualizes these findings. Tepanosyan, et al.^96^ conducted a similar assessment in a kindergarten in Yerevan, where HI values in children surpassed acceptable thresholds, aligning with the heightened sensitivity observed in Sanandaj’s child population. Additionally, Zhang, et al.^97^ identified ingestion, inhalation, and dermal contact as the primary non-carcinogenic risk factors for PTEs in the Green Land of China, while Ghanavati, et al.^22^ emphasized the increased sensitivity of children to non-carcinogenic risks posed by PTEs in urban soils. These studies highlight the consistent pattern of higher vulnerability in children, necessitating targeted risk mitigation strategies.

Furthermore, the differential risk levels between adults and children highlight the need for age-specific risk assessment and management strategies. Children’s developing physiological systems make them more susceptible to the toxic effects of PTEs, necessitating stricter regulatory standards and more aggressive remediation efforts in areas frequented by younger populations, such as playgrounds and schools. The presence of elevated CR values for carcinogenic metals also suggests a need for long-term epidemiological studies to monitor cancer incidence rates in the affected populations, thereby providing a clearer picture of the public health implications over time.

The sources of these PTEs in Sanandaj’s surface soil are multifaceted, encompassing industrial emissions, vehicular traffic, and inadequate waste management practices. Industrial activities such as metal plating, battery manufacturing, non-ferrous metal processing, and vehicular emissions significantly release Zn, Cu, Ni, Cr, and Pb into the environment. Specifically, galvanization processes and stainless-steel production are significant contributors to Zn, Cr, and Ni contamination. At the same time, vehicular traffic is a primary source of Cu and Pb through brake wear and tire degradation. Additionally, improper disposal and management of industrial and municipal waste can lead to Cd leaching into the soil, further exacerbating contamination levels. These anthropogenic sources are compounded by possible geological factors, such as the natural presence of certain metals in the region’s bedrock, which may contribute to baseline contamination levels^11,31,94^.

Addressing these health risks requires a multifaceted approach. Implementing enhanced monitoring through comprehensive soil tracking programs will enable continuous assessment of PTE concentrations and the identification of pollution hotspots for timely intervention^2,11,98^. Stricter industrial regulations and robust waste management practices within critical industrial sectors are essential to minimize the release of PTEs into the environment. Promoting cleaner vehicle technologies, improving public transportation infrastructure, and implementing traffic reduction measures can significantly reduce vehicular emissions contributing to soil contamination. Additionally, employing effective soil remediation techniques such as phytoremediation, soil washing, and stabilization can decrease the bioavailability of PTEs and mitigate soil contamination^60,71,98^. Increasing community awareness regarding the sources and risks of PTEs pollution is crucial for encouraging environmentally friendly practices and supporting sustainable development initiatives. Developing localized environmental policies tailored to Sanandaj’s specific PTE profile will ensure effective management and reduction of PTEs pollution, safeguarding ecosystem integrity and public health^23,67,97^.

In conclusion, the human health risk assessment in Sanandaj city reveals that while non-carcinogenic risks from PTEs remain low, the carcinogenic risks posed by chromium, arsenic, nickel, cadmium, and lead are significantly above acceptable limits for both adults and children. These findings highlight the urgent need for targeted mitigation measures to protect vulnerable populations, particularly children, from the adverse health effects of PTEs contamination in urban soils. Comparative analysis with other metropolitan areas underscores the critical role of industrial activities, regulatory frameworks, and environmental management practices in influencing PTE concentrations. Addressing these risks through enhanced monitoring, stringent industrial regulations, effective remediation strategies, and public education is essential to protect the health and well-being of Sanandaj’s residents, ensuring a sustainable and healthy environment.

**Table 7:** Carcinogenic and non-carcinogenic risk of studied PTEs.

PTEs	HQ_Ingestion_		HQ_inhalation_		HQ_dermal_		Hazard index (HI)		Cancer risk (CR)	
	Adults	Children	Adults	Children	Adults	Children	Adults	Children	Adults	Children
Zn	2.1 × 10^−4^	1.6 × 10^−3^	2.02 × 10^−8^	4.5 × 10^−8^	6.5 × 10^−6^	2.5 × 10^−6^	7.3 × 10^−4^	5.3 × 10^−3^	-	-
Cu	9.7 × 10^−5^	7.2 × 10^−4^	9.1 × 10^−9^	2 × 10^−8^	2.9 × 10^−6^	1.1 × 10^−6^	2.6 × 10^−3^	1.8 × 10^−2^	-	-
Ni	9.6 × 10^−5^	7.1 × 10 − 4	9.07 × 10^−9^	2 × 10^−8^	2.9 × 10^−6^	1.1 × 10^−6^	5.3 × 10^−3^	3.6 × 10^−2^	4.5 × 10^−3^	3 × 10^−2^
Pb	4.6 × 10^−5^	3.4 × 10^−4^	4.3 × 10^−9^	9.6 × 10^−9^	1.4 × 10^−6^	5.5 × 10^−7^	1.5 × 10^−2^	9.9 × 10^−2^	6.6 × 10^−4^	4.1 × 10^−3^
Cr	1.9 × 10^−4^	1.4 × 10^−3^	1.7 × 10^−8^	3.9 × 10^−8^	5.8 × 10^−6^	2.2 × 10^−6^	1.6 × 10^−1^	5.1 × 10^−1^	6.7 × 10^−6^	2.16 × 10^−5^
Cd	4.1 × 10^−7^	3 × 10^−6^	3.8 × 10^−11^	8.6 × 10^−11^	1.2 × 10^−8^	4.9 × 10^−9^	1.6 × 10^−3^	3.5 × 10^−3^	1 × 10^−2^	2.2 × 10^−2^
As	7.9 × 10^−6^	5.9 × 10^−5^	7.4 × 10^−10^	1.6 × 10^−9^	2.4 × 10^−7^	9.4 × 10^−8^	4.2 × 10^−2^	2 × 10^−1^	6.3 × 10^−2^	3 × 10^−1^

### PAH concentration in surface soils of Sanandaj City

The concentrations of Σ15PAHs in the 53 soil samples collected from Sanandaj’s urban areas ranged from 126.44 to 2460.87 µg/kg, with a mean value of 850.81 µg/kg, as determined by GC-MS analysis. This wide range reflects significant variability in PAH contamination, with elevated concentrations (e.g., 2460.87 µg/kg in sample S2) indicating strong anthropogenic inputs from vehicular emissions, industrial activities, and combustion sources in urban hotspots, while lower values (e.g., 126.44 µg/kg in sample S6) are associated with less impacted residential areas. The mean concentration of 850.81 µg/kg, highlighting the influence of urban activities on soil contamination in Sanandaj. Detailed concentration profiles for individual PAHs, including 3-ring, 4-ring, and 5-ring compounds, are provided in Supplementary Table S3, with quality control data (surrogate recoveries: 85%–105%, RSD < 10%) ensuring analytical reliability. These findings underscore the need for targeted environmental monitoring and remediation efforts in high-risk urban zones. Among the individual PAHs, Benzo[b]fluoranthene (BbF) exhibited the highest average concentration at 51.356 µg/kg, while Benzo[a]pyrene (BaP) and Benzo[ghi]perylene (BghiP) were below the detection limit of the analytical instrument, indicating minimal presence in the sampled soils^12,14^.

The descending order of average PAH concentrations in the surface soils of Sanandaj is BbF > Anthracene (Ant) > Phenanthrene(Phe) > Benzo[k]fluoranthene(BkF) > Benzo[a]anthracene (BaA) > Dibenz[a, h]anthracene (DBA) > Fluorene (Flu) > Fluoranthene (Flt) > Indeno[1,2,3-cd]pyrene (InP) > Pyrene (Pyr) > Acenaphthylene (Acy) > Acenaphthene (Ace) > BaP = BghiP. This hierarchy underscores the predominance of specific PAH compounds, particularly those associated with combustion processes^60,87,99^.

The carcinogenic PAHs (CANPAHs) ratio to the total PAHs (ΣPAHs) ranges from 21.0 to 57.0 µg/kg. The ratio of non-carcinogenic PAHs (NCANPAHs) to ΣPAHs varies between 40.0 and 41.0 µg/kg. These ratios indicate a substantial presence of carcinogenic PAHs in the soil, posing potential health risks. Furthermore, the analysis distinguishes between low molecular weight (LMW) PAHs, comprising 53.33% of the total PAHs with an average concentration of 31.285 µg/kg, and high molecular weight (HMW) PAHs, accounting for 47.66% of the total PAHs with an average concentration of 5.565 µg/kg. The significant proportion of HMW PAHs suggests a predominance of pyrogenic sources, such as vehicular emissions and industrial combustion processes.

The higher concentration of HMW PAHs like BbF and BkF aligns with emissions from diesel engines and industrial activities, which are rich in high molecular weight PAHs. This is further corroborated by the elevated skewness values observed for several PAHs, indicating a right-skewed distribution typical of contamination from point sources. The presence of HMW PAHs in substantial amounts points to ongoing anthropogenic activities contributing to soil contamination, necessitating targeted pollution control measures.

The toxic equivalence (TEQ) of PAHs, calculated based on Toxic Equivalency Factors (TEFs), averaged 83.17 µg/kg, indicating the potentially toxic impact of the PAH mixture in the soil. The TEQ/ΣPAHs ratio, averaging 10%, signifies that a significant portion of the total PAHs contributes to the overall toxic risk. This metric is critical in understanding PAH contamination’s potential ecological and health impacts, as it accounts for the varying toxicities of individual PAHs.

Comparatively, the PAH concentrations in Sanandaj’s surface soils are influenced by local industrial activities, vehicular traffic, and possibly geological factors, similar to other urban environments. However, the specific profile, characterized by a higher prevalence of HMW PAHs, suggests a dominant influence of pyrogenic sources over petrogenic ones. This distinction is essential for developing targeted remediation strategies, as pyrogenic PAHs often require different treatment approaches than petrogenic PAHs.

In conclusion, the PAH concentration analysis in Sanandaj city’s surface soils reveals a significant presence of both carcinogenic and non-carcinogenic PAHs, predominantly of pyrogenic origin. The high BbF and other HMW PAHs point to ongoing emissions from industrial and vehicular sources, underscoring the need for stringent environmental controls and remediation efforts to mitigate the health and ecological risks associated with PAH contamination in urban soils.

This comprehensive analysis of PAH concentrations in Sanandaj city’s surface soils highlights the prevalence of carcinogenic and non-carcinogenic PAHs, predominantly from pyrogenic sources such as vehicular emissions and industrial combustion processes. The high levels of BbF and other HMW PAHs suggest significant contributions from diesel engine exhaust and industrial activities, aligning with the observed skewness values that indicate point source pollution. The substantial presence of carcinogenic PAHs (CANPAHs) underscores the potential health risks, particularly given the high TEQ values relative to the total PAH concentration. These findings emphasize the urgent need for targeted pollution control measures and remediation strategies to mitigate PAH contamination’s adverse environmental and health impacts in Sanandaj’s urban soils.

### Environmental risk assessment of PAHs

To evaluate the ecotoxicological impact of polycyclic aromatic hydrocarbons (PAHs) in the surface soils of Sanandaj city, this study utilized the Effects Range Low (ERL) and Effects Range Median (ERM) benchmarks^54^. The ERL serves as the lower threshold below which adverse biological effects are unlikely to occur, while the ERM represents the median threshold above which significant biological impacts are expected.

According to Table 8, the average concentrations of most PAH compounds-namely Naphthalene (Nap), Acenaphthylene (Acy), Acenaphthene (Ace), Phenanthrene (Phe), Fluorene (Flu), Fluoranthene (Flt), Pyrene (Pyr), Benz[a]anthracene (BaA), and Dibenz[a, h]anthracene (DBA)-are below their respective ERL values^99,100^. This indicates that these PAHs are at levels unlikely to cause biological harm under current environmental conditions in Sanandaj. However, Anthracene (Ant) and Fluorene (Flu) exhibit concentrations that exceed their ERL values but remain below their ERM values. This suggests that while these PAHs are generally below the levels of concern, there is a potential for occasional biological side effects in areas where their concentrations approach the ERM thresholds.

The distribution of PAH concentrations further reveals that high molecular weight (HMW) PAHs constitute a significant portion of the total PAHs, accounting for 47.66% with an average concentration of 5.565 µg/kg. This predominance of HMW PAHs, such as BbF and BkF, indicates pyrogenic sources, including vehicular emissions and industrial combustion processes. The substantial presence of these HMW PAHs aligns with the observed skewness values, which suggest a right-skewed distribution typical of point source pollution.

The environmental risk assessment demonstrates that the majority of PAH compounds in the surface soils of Sanandaj city are present at concentrations below the ERL, indicating minimal ecological threat. However, the elevated levels of Anthracene and Fluorene, though still below their ERM thresholds, highlight a potential for occasional biological impacts. The significant proportion of high molecular weight PAHs underscores the influence of combustion-related sources on soil contamination. These findings emphasize the necessity for ongoing monitoring and implementing targeted pollution control measures to mitigate the ecological effects of PAH contamination, particularly from vehicular and industrial emissions. By addressing these sources, Sanandaj can better safeguard soil health and protect the surrounding ecosystems from the adverse impacts of PAH pollutants.

**Table 8:** Standard contamination criteria of PAHs in surface soil samples of Sanandaj city.

PAH Compounds	SQG	S	Sanandaj Surface Soil	
	ERL	ERM	Average	Max
	µg/kg		µg/kg	
Nap	160	2100	4.46	12.13
Acy	44	640	9.2	33.38
Ace	16	500	2.25	12.1
Flu	19	540	27.53	67.37
Phe	240	1500	100.85	234.31
Ant	85.3	1100	141.02	476.35
Flt	600	5100	21.89	85.29
Pyr	665	2600	12.61	96.47
BaA	261	1600	60.7	162.16
BaP	430	1600	N.D	N.D
DBA	63.4	260	31.65	35.34

### Cancer risk assessment

A comprehensive health risk assessment was conducted to evaluate the carcinogenic risks posed by polycyclic aromatic hydrocarbons (PAHs) in the surface soils of Sanandaj City. This assessment focused on three primary exposure pathways: ingestion, dermal absorption, and inhalation, targeting children and adults^64^. Utilizing Toxic Equivalency Factors (TEFs) for the 15 studied PAHs, the total Benzo[a]pyrene equivalent (BaPeq) concentrations were calculated based on specific TEF values as outlined by^101^. The results, summarized in Table 9, reveal distinct exposure patterns and associated cancer risks for different demographic groups.

The analysis indicated that dermal absorption emerged as the most significant exposure pathway for children and adults, followed by ingestion and inhalation. Specifically, children exhibited higher Incremental Lifetime Cancer Risk (ILCR) values through ingestion (1.8 × 10^−4^) and dermal contact (2.25 × 10^−4^) compared to adults (1.41 × 10^−4^) for ingestion and 2.51 × 10^−4^ for dermal contact). Inhalation posed a negligible risk for both groups, with ILCR values significantly below the threshold of concern (3.51 × 10^−9^) for children and 1.09 × 10^−8^ for adults).

The lifetime cancer risk (CR) associated with PAH exposure was calculated to be 4.06 × 10^−4^ for children and 3.92 × 10^−4^ for adults. According to the United States Environmental Protection Agency (US EPA) standards, CR values below 1 × 10^−6^ are considered negligible, values between 1 × 10^−6^ and 1 × 10^−4^ indicate potential carcinogenic risks, and values exceeding 1 × 10^−4^ suggest high carcinogenic risk^59,95^. In this study, children and adults in Sanandaj City have CR values surpassing the acceptable limits, categorizing the carcinogenic risk as high for both populations. Notably, children face a slightly higher risk than adults, underscoring their increased vulnerability to PAH exposure.

Children are inherently more susceptible to PAH exposure due to several factors. Their behavioral patterns, such as increased hand-to-mouth activities, lead to higher ingestion rates of contaminated soil. Additionally, children’s developing nervous and immune systems heighten their physiological susceptibility to the toxic effects of carcinogens^12,101,102^. Furthermore, children consume more soil particles per kilogram of body weight than adults, amplifying their exposure levels cumulative.

High molecular weight PAHs, such as Benzo[b]fluoranthene (BbF) and Benzo[k]fluoranthene (BkF), indicate significant contributions from diesel engine exhaust and industrial activities. These sources release substantial quantities of carcinogenic PAHs into the environment, leading to elevated concentrations in urban soils. These high molecular weight PAHs align with the observed skewness values, suggesting a right-skewed distribution typical of point source pollution.

The elevated CR values for carcinogenic PAHs in Sanandaj’s surface soils pose severe health risks. This situation is further aggravated by the high molecular weight PAHs, which are more persistent in the environment and pose more significant health risks due to their bioaccumulation ability^28^. Chromium (Cr) and nickel (Ni) are recognized carcinogens capable of contaminating groundwater and entering the food chain, thereby increasing the likelihood of respiratory cancers such as lung cancer. Arsenic (As) and cadmium (Cd), even at lower concentrations, are highly toxic and can accumulate in biological systems, leading to chronic conditions like kidney damage and various cancers. Lead (Pb) exposure is particularly detrimental to children, impairing neurological development and resulting in cognitive and behavioral issues.

Addressing these elevated cancer risks requires a multifaceted approach. Implementing enhanced monitoring through comprehensive soil tracking programs will enable continuous assessment of PAH concentrations and the identification of pollution hotspots for timely intervention. Stricter industrial regulations and robust emission controls within key sectors are essential to minimize the release of PAHs into the environment. Promoting cleaner vehicle technologies, improving public transportation infrastructure, and implementing traffic reduction measures can significantly reduce vehicular emissions contributing to soil contamination. Additionally, employing effective soil remediation techniques such as phytoremediation, soil washing, and stabilization can decrease the bioavailability of PAHs and mitigate soil contamination. Increasing community awareness regarding the sources and risks of PAH pollution is crucial for encouraging environmentally friendly practices and supporting sustainable development initiatives. Developing localized environmental policies tailored to Sanandaj’s specific PAH profile will ensure effective management and reduction of PTEs and PAH pollution, safeguarding ecosystem integrity and public health.

In conclusion, the cancer risk assessment of PAHs in the surface soils of Sanandaj City reveals that both children and adults are exposed to carcinogenic PAHs at levels exceeding safe thresholds. Children, in particular, face higher risks due to their behavioral and physiological susceptibilities. The predominant presence of high molecular weight PAHs points to vehicular and industrial sources as major contributors to soil contamination. Addressing these risks through enhanced monitoring, stringent emission controls, effective remediation strategies, and comprehensive public health initiatives is essential to protect the health and well-being of Sanandaj’s residents, ensuring a sustainable and healthy environment.

**Table 9:** Concentration of PAHs in surface soil of Sanandaj city.

	Average	Min	Max
TEQ	73.55	25.81	199.03
Children			
ILCRs ing	1.8 × 10^−4^	6.3 × 10^−5^	4.9 × 10^−4^
ILCRs der	2.25 × 10^−4^	7.9 × 10^−5^	6.1 × 10^−4^
ILCRs inh	3.51 × 10^−9^	1.23 × 10^−9^	9.5 × 10^−4^
Cancer risk	4.0610^−4^	1.42 × 10^−4^	1.1 × 10^−3^
Adults			
ILCRs ing	1.41 × 10^−4^	4.96 × 10^−5^	3.8 × 10^−4^
ILCRs der	2.51 × 10^−4^	8.81 × 10^−5^	6.7 × 10^−4^
ILCRs inh	1.09 × 10^−8^	3.84 × 10^−9^	2.96 × 10^−8^
Cancer risk	3.92 × 10^−4^	1.37 × 10^−4^	1.06 × 10^−3^

The Incremental Lifetime Cancer Risk (ILCR) from ingestion in children is more significant than in adults due to their hand-to-mouth behaviors, which facilitate the intake of contaminated soil^12,28^. Conversely, the risks linked to dermal contact and inhalation are more pronounced in adults. This can be attributed to the larger surface area (SA) and more prolonged exposure duration (ED) typical of adults^103^. Additionally, the ongoing development of children’s nervous and immune systems can heighten their vulnerability to carcinogenic substances^29,57,100^. Consequently, the potential health risks for children exposed to PAHs in contaminated soil are significantly higher than for adults^22^.

ILCR values below 1 × 10^−6^ are deemed negligible, while values between 1 × 10^−6^ and 1 × 10^−4^ suggest a potential carcinogenic risk. Values exceeding 1 × 10^−4^ indicate a significant potential for carcinogenic risk^104,105^. In this study, the overall cancer risk (CR) calculated for children is 4.06 × 10^−4^, while for adults, it is 3.92 × 10^−4^. Both values surpass the acceptable threshold of 1 × 10^−6^ for both age groups. Given that the total cancer risk exceeds 1 × 10^−4^, it signifies a high potential for carcinogenic risk, particularly for children compared to adults^22^.

### Potential ecosystem risk

**Fig. 2:**
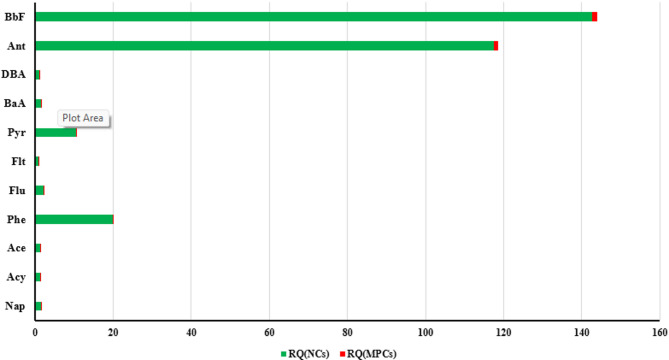
Stacked bar chart showing Risk Quotients (RQ(NCs) and RQ(MPCs)) for 13 priority PAHs in Sanandaj’s surface soils. High RQ values for Anthracene and Benzo[b]fluoranthene indicate significant ecological risks requiring remediation.

The ecological risk posed by Polycyclic Aromatic Hydrocarbons (PAHs) in the surface soils of Sanandaj city was assessed using Risk Quotients (RQs) calculated for 53 soil samples, based on Negligible Concentrations (NCs) and Maximum Permissible Concentrations (MPCs). Table 10; Fig. 2 presents the average RQ(NCs) and RQ(MPCs) values for 13 priority PAHs, along with their Toxic Equivalency Factors (TEFs), NCs, and MPCs, sourced from in^22,52^. The risk classification follows established criteria: RQ(NCs) < 1 indicates negligible risk; RQ(NCs) ≥ 1 and RQ(MPCs) < 1 suggests low to moderate risk; and RQ(MPCs) ≥ 1 indicates high risk requiring remediation^22,52^.

For Fluoranthene (Flt) and Indeno[1,2,3-cd]pyrene (InP), both RQ(NCs) and RQ(MPCs) values were below one (e.g., Flt: RQ(NCs) = 0.8, RQ(MPCs) = 0.008; InP: RQ(NCs) = 0.3, RQ(MPCs) = 0.003), indicating negligible ecological risk. Similarly, compounds such as Naphthalene (Nap), Acenaphthylene (Acy), Acenaphthene (Ace), Phenanthrene (Phe), and Pyrene (Pyr) exhibited RQ(MPCs) values below one, suggesting low ecological risk. However, their RQ(NCs) values exceeded one (e.g., Phe: RQ(NCs) = 19.8; Pyr: RQ(NCs) = 10.5), indicating moderate environmental risk due to their presence in urban soils. In contrast, Anthracene (Ant) and Benzo[b]fluoranthene (BbF) showed RQ(NCs) and RQ(MPCs) values greater than one (e.g., Ant: RQ(NCs) = 117.5, RQ(MPCs) = 1.175; BbF: RQ(NCs) = 142.6, RQ(MPCs) = 1.426), highlighting a high ecological risk in Sanandaj’s surface soils.

For ΣPAHs, the average RQ(NCs) was 363.1, indicating moderate risk, while RQ(MPCs) was 3.663, suggesting high risk across the sampled sites. These findings are consistent with urban soil studies and align with Aydın et al. (2023), who reported elevated PAH risks in coastal sediments due to high molecular weight compounds like BbF [108]. The moderate to high ecological risk in Sanandaj, particularly from Ant and BbF, underscores the need for targeted environmental monitoring and remediation strategies to mitigate potential impacts on soil ecosystems. Detailed concentration data and RQ calculations for individual PAHs are provided in Supplementary Tables S3 and S4, complementing the selected PAH risk profiles in Table 10.

**Table 10:** Average values of RQ(_NCS_) and RQ(_MPCS_) in aromatic compounds in surface soil of Sanandaj City (µg/kg).

	TEF	NC_S_	MPC_S_	RQ_(NCS)_	RQ_(MPCS)_	Risk Classification
		(µg/kg)	(µg/kg)	(µg/kg)	(µg/kg)	
Naphthalene (Nap)	0.001	1.4	140	3.2	0	Low to Moderate
Acenaphthene (Acy)	0.001	1.2	120	7.7	0.077	Low to Moderate
Acenaphthene (Ace)	0.001	1.2	120	1.9	0.019	Low to Moderate
Fluorene (Flu)	0.001	1.2	120	22.9	0.229	Low to Moderate
Phenanthrene (Phe)	0.001	5.1	510	19.8	0.198	Low to Moderate
Anthracene (Ant)	0.01	1.2	120	117.5	1.175	High
Benzo(b)fluoranthene (Flt)	0.01	26	2600	0.8	0.008	Negligible
Pyrene (Pyr)	0.001	1.2	120	10.5	0.105	Low to Moderate
Benz(a)anthracene (BaA)	0.001	2.5	250	24.3	0.243	Low to Moderate
Benzo(b)fluoranthene (B(b)f))	0.1	2.5	250	142.6	1.426	High
Benzo(k)fluoranthene (B(k)f))	0.1	24	2400	2.6	0.026	Low to Moderate
Indeno[1,2,3-cd]pyrene (Inp)	0.1	59	5900	0.3	0.003	Negligible
Dibenzo(a, h)anthracene (DBA)	1	2.6	260	12.2	0.122	Low to Moderate
ΣPAH_s_				363.1	3.663	High

### Isotopic analysis

Lead is characterized by four isotopes: ^204^Pb, ^206^Pb, ^207^Pb, and ^208^Pb. These isotopes result from 238U, 235U, and 232Th radioactive decay, except ^204^Pb. Their frequencies vary depending on their respective sources. Different anthropogenic sources and ore minerals display unique isotopic traits^74^. The isotopic ratios of lead, aside from ^204^Pb, remain consistent throughout industrial or environmental/anthropogenic processes^82^. Therefore, isotopic ratios serve as valuable indicators for identifying sources and tracing the pathways of lead in pollution studies^70^.

**Fig. 3:**
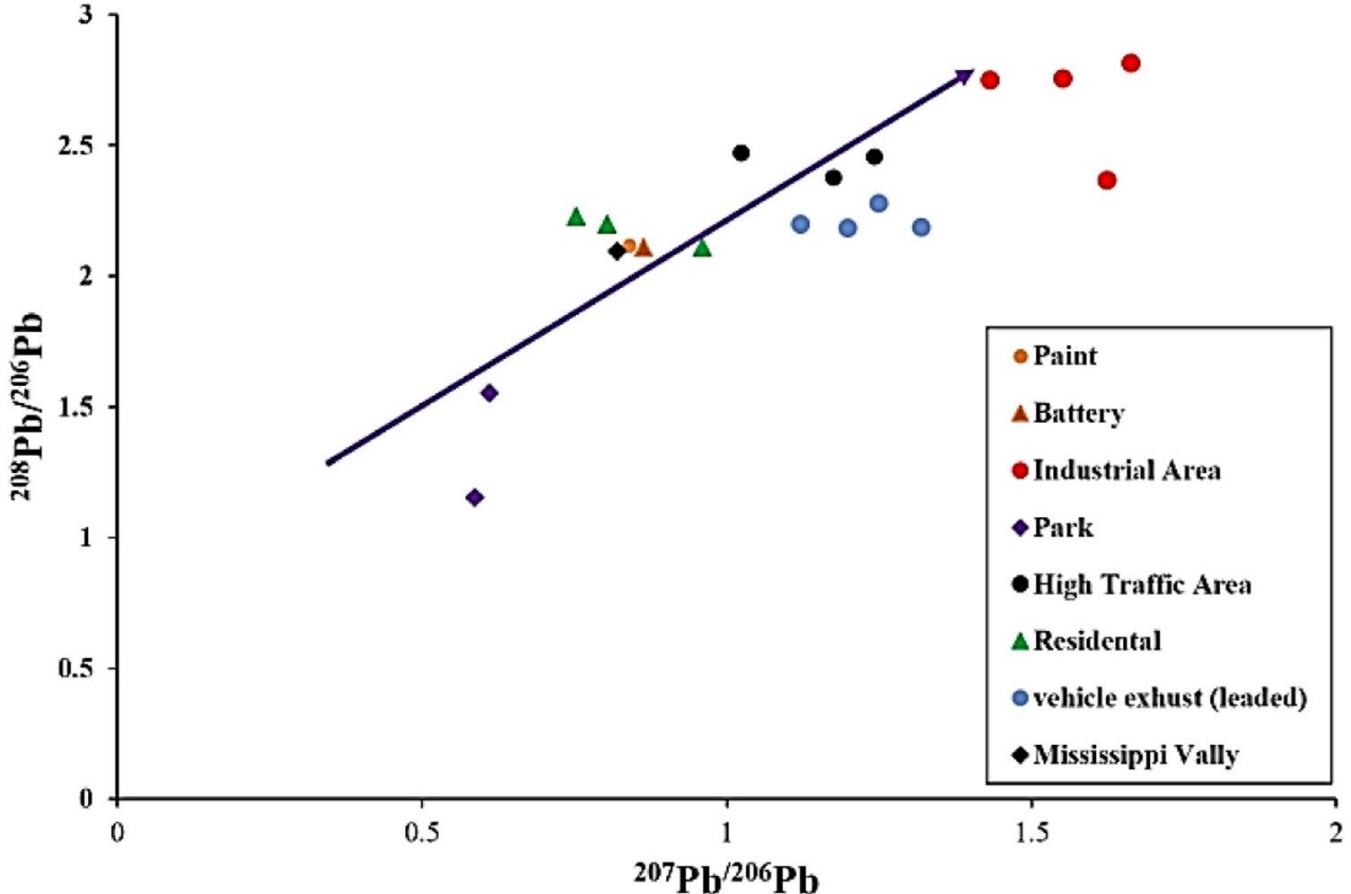
Plots of ^208Pb^/^206^Pb versus ^206^Pb/^207^Pb in the Sanandaj urban soil and the known sources.

Table S4 presents the isotopic ratios of Pb for 12 surface soil samples. The findings indicate that samples collected from industrial areas display unique isotopic compositions compared to the remaining samples. Generally, soil samples affected by pollution exhibit lower ratios of ^206^Pb/^204^Pb, ^207^Pb/^204^Pb, and ^208^Pb/^204^Pb, along with higher ^208^Pb/^206^Pb ratios^106^. These trends are consistent with the calculated ratios observed in the collected samples, as detailed in Table S4 and illustrated in Fig. 3.

The isotopic ratios of Pb in uncontaminated soils within the studied region, exemplified by S6 and S7, exhibit a more radiogenic nature, with values akin to the average ratio found in the continental crust (^206^Pb/^207^Pb=1.20)^72^. Consequently, these samples can be classified as natural soils with geogenic isotopic ratios rather than deriving from anthropogenic sources. In contrast, the lower ^206^Pb/^204^Pb ratios observed in samples surrounding industrial areas (including S1, S2, S3, and S4) point to the presence of anthropogenic sources of Pb in the surface soils^107^. The isotopic compositions of other Pb isotopes suggest a combination of anthropogenic and geogenic sources^108^.

The scatter plot illustrating the correlation between ^208^Pb/^206^Pb and ^208^Pb/^206^Pb ratios reveals a complex and non-linear pattern across various sampling sites, indicating distinctive characteristics of Pb isotopes within the studied area. Furthermore, Fig. 2 presents compelling evidence that samples originating from industrial areas (S1, S2, S3, and S4) exhibit significantly higher isotopic ratios than other land uses in the study area and leaded exhaust emissions from vehicles. These heightened isotopic ratios observed in industrial samples indirectly suggest the involvement of active companies located in the industrial town as contributors to Pb pollution.

Interestingly, the samples from the park (S6 and S7) showcase two distinct isotopic ratios. This implies a potential influence of industrial activities on the isotopic composition of Pb in the surrounding environment. Additionally, samples collected from a residential area with a high traffic volume (such as S11 and S12) are scattered near areas with leaded vehicle exhaust, displaying isotopic ratios resembling those found in Mississippi ore. This finding strongly indicates the impact of traffic-related pollution in Sanandaj, particularly concerning Pb contamination.

It’s noteworthy that sample A9, located at the intersection of the main square and the ring road of Sanandaj city, demonstrates higher isotopic ratios than other residential samples. This site undergoes a substantial traffic influx, contributing to heightened levels of Pb pollution.

The isotopic analysis offers valuable insights into the sources and distribution of Pb contamination in the studied area. It underscores the intricate interplay between industrial activities, traffic emissions, and natural influences on the isotopic composition of Pb in the environment.

Various equations and formulas have been proposed to distinguish between different sources of Pb, whether they are naturally occurring (geogenic) or caused by human activities (anthropogenic).

Isotopic analysis is a powerful tool for identifying and quantifying the sources of soil contamination. By examining the ratios of different isotopes of PTEs, such as lead (Pb), researchers can distinguish between various contamination sources and determine their contributions to overall contamination levels. In this study, we utilize the isotopic ratios $\:\frac{\mathrm{P}\mathrm{b}206}{\mathrm{P}\mathrm{b}207}\:\:$and $\:\frac{\mathrm{P}\mathrm{b}208}{\mathrm{P}\mathrm{b}207}$​ to assess the origins of Pb contamination in soil samples. Additionally, isotopic mixing models are applied to quantify the contributions of multiple contamination sources, specifically geogenic sources (F1), industrial emissions (F2), and vehicle traffic (F3).

Isotopic mixing models are mathematical frameworks used to deconvolute the contributions of multiple contamination sources to a single sample’s isotopic composition. In this study, a three-end-member mixing model is employed to determine the proportions of geogenic sources (F1), industrial emissions (F2), and vehicle traffic (F3) contributing to Pb contamination in soil samples. The model integrates Pb concentration and isotopic ratios as crucial parameters for accurate source apportionment (Table 11).

F1, F2, and F3 represent the fractional contributions of geogenic sources, industrial emissions, and vehicle traffic to the total Pb contamination in the soil sample, respectively^82^. These fractions satisfy the constraint given by the following Eq. (19):

19 $$\:\mathrm{F}1+\mathrm{F}2+\mathrm{F}3=1$$

#### $\:{R}_{1}={\frac{\mathrm{P}\mathrm{b}206}{\mathrm{P}\mathrm{b}207}}_{source1}=1.2252$

isotopic ratio of Pb for geogenic sources.

#### $\:{R}_{1}={\frac{\mathrm{P}\mathrm{b}206}{\mathrm{P}\mathrm{b}207}}_{source2}$

isotopic ratio of Pb for industrial sources (ranging from 1.1427 to 1.1567).

#### $\:{R}_{1}={\frac{\mathrm{P}\mathrm{b}206}{\mathrm{P}\mathrm{b}207}}_{source3}=1.097$

isotopic ratio of Pb for vehicle sources.

C1: 6681 mg/kg: concentration of Pb in geogenic sources.

C2: 2397 mg/kg: concentration of Pb in industrial sources.

C2: 2397 mg/kg: concentration of Pb in vehicle traffic sources.

C_soil_: concentration of Pb in soil sample.

$\:{R}_{soil}={\frac{\mathrm{P}\mathrm{b}206}{\mathrm{P}\mathrm{b}207}}_{sample}:$ Isotope ratio $\:{\frac{\mathrm{P}\mathrm{b}206}{\mathrm{P}\mathrm{b}207}}_{sample}$in the soil sample.

#### $\acute{{R}}_{soil}={\frac{\mathrm{P}\mathrm{b}208}{\mathrm{P}\mathrm{b}207}}_{sample}$

Isotope ratio $\:{\frac{\mathrm{P}\mathrm{b}208}{\mathrm{P}\mathrm{b}207}}_{sample}$in the soil sample.

The following set of linear equations (Eqs. 20–22) defines the isotopic mixing model for three contamination sources:

20 $$\:\mathrm{F}1+\mathrm{F}2+\mathrm{F}3=1$$

21 $$\:\mathrm{F}1.\mathrm{R}1+\mathrm{F}2.\mathrm{R}2+\mathrm{F}3.\mathrm{R}3={R}_{soil}$$

22 $$\:\frac{F1}{C1}+\frac{F2}{C2}+\frac{F3}{C3}=\frac{1}{{C}_{soil}}$$

Where:

F1​: Fractional contribution of geogenic sources.

F2: Fractional contribution of industrial emissions.

F3: Fractional contribution of vehicle traffic.

These equations ensure that the soil sample’s isotopic composition and Pb concentration are represented as weighted averages of the contributions from each contamination source. Based on literature and regional industrial activities, three primary contamination sources were defined with their respective isotopic signatures and Pb concentrations:

#### Source 1: geogenic sources

R1​=1.2252

C1​=6681 mg/kg.

#### Source 2: industrial emissions

R2​ ranges from 1.1427 to 1.1567.

C2​=2379 mg/kg.

#### Source 3: vehicle traffic

R3​=1.097

C3​=2379 mg/kg.

The isotopic mixing model results, as presented in Table 11, reveal distinct contributions of geogenic sources (F1), industrial emissions (F2), and vehicle traffic (F3) to lead (Pb) contamination across the twelve soil samples.

Geogenic Sources (F1) contribute significantly to Pb contamination in samples S1, S2, S4, S5, S6, S7, S8, S9, S10, S11, and S12, with their contributions ranging from 10% to 45%. The mean contribution of geogenic sources is approximately 24.2%, which aligns well with values reported in existing literature. This consistency underscores the substantial role of natural geological backgrounds in influencing Pb levels within the soil environment.

Industrial Emissions (F2) emerge as the dominant source of Pb contamination in most samples, contributing between 40% and 70%, with an average contribution of 38.5%. This significant impact highlights the pervasive influence of industrial activities on Pb pollution in the study area. The high F2 values are likely attributable to numerous manufacturing facilities and associated industrial processes, which release substantial amounts of Pb into the environment.

**Fig. 4:**
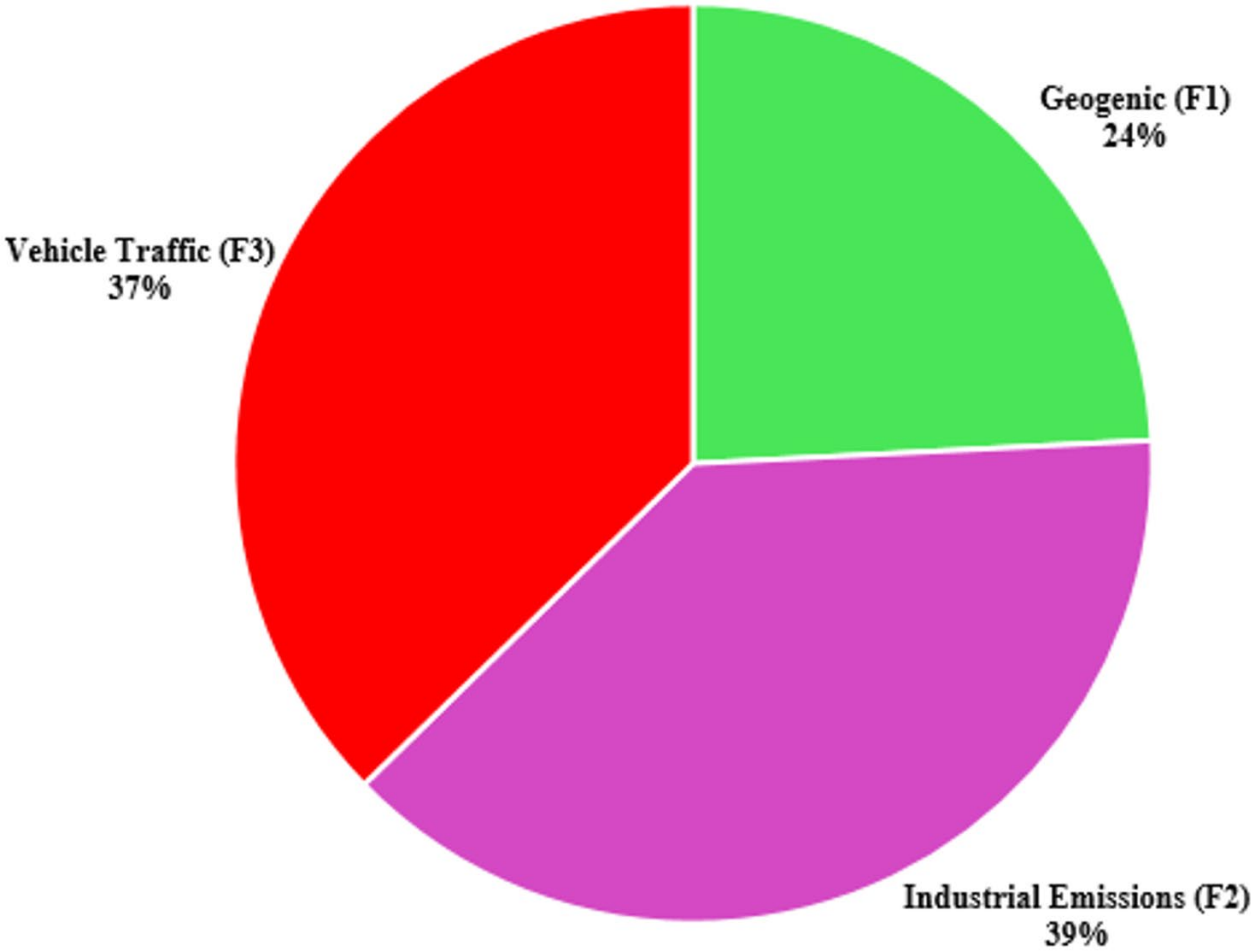
Pie chart depicting the contributions of geogenic, industrial, and vehicle traffic sources to Pb contamination in soil sample S2 from Sanandaj. Industrial emissions (38.5%) and vehicle traffic (37.3%) dominate over geogenic sources (24.2%).

Vehicle Traffic (F3) also exhibits notable contributions across multiple samples, ranging from 15% to 30%, with a mean value of 37.3%. This substantial influence emphasizes the role of vehicular emissions in Pb contamination, reflecting the high volume of traffic and related exhaust emissions within the region. The consistent presence of vehicle traffic as a major contributor aligns with studies identifying vehicular emissions as a key anthropogenic source of Pb pollution^34,70,82^. The dominance of industrial (38.5%) and vehicular (37.3%) Pb sources in sample S2 (Fig. 4), located in a traffic-heavy area, aligns with Sanandaj’s land use-driven contamination (Supplementary Table S4).

Overall, the combined contributions of industrial emissions and vehicle traffic constitute the primary sources of Pb pollution in the studied area, while geogenic sources also play a considerable role. These findings are consistent with existing research, which frequently identifies industrial and vehicular activities as major contributors to environmental Pb contamination. The significant variability in the contribution factors (F indices) across different land use sampling sites further indicates the heterogeneous nature of Pb pollution sources, influenced by localized industrial activities and traffic density^82^.

Figure 4; Table 11 summarizes the contributions of Pb sources in soil samples, illustrating the prominent roles of industrial emissions and vehicle traffic alongside geogenic sources^82^. The predominance of anthropogenic sources in Pb contamination underscores the need for targeted environmental management and regulatory measures to mitigate Pb pollution from industrial and vehicular activities^82^.

**Table 11:** Contributions of Pb sources in soil Samples.

Sample	F1 (Geogenic)	F2 (Industrial)	F3 (Traffic)
S1	0.3	0.5	0.2
S2	0.25	0.5	0.25
S3	0.1	0.6	0.3
S4	0.35	0.45	0.2
S5	0.2	0.6	0.2
S6	0.4	0.4	0.2
S7	0.45	0.4	0.15
S8	0.15	0.55	0.3
S9	0.35	0.45	0.2
S10	0.1	0.7	0.2
S11	0.25	0.5	0.25
S12	0.2	0.5	0.3

### Comparison with previous research

The contributions of Pb sources identified in this study are consistent with findings from previous research, reinforcing the predominant role of anthropogenic activities in Pb contamination within urban and industrialized regions^82,109,110^. Specifically, Industrial Emissions (F2) demonstrate a contribution range of 40% to 70%, with a mean value of 38.5%, corroborating the significant impact of industrial activities on Pb pollution as highlighted by^111^. This substantial influence is likely attributable to numerous manufacturing facilities and associated industrial processes in the study area, which release considerable amounts of Pb into the environment.

Similarly, Vehicle Traffic (F3) exhibits significant contributions across multiple samples, ranging from 15% to 30%, with a mean value of 37.3%. This underscores the substantial impact of vehicular emissions on Pb contamination, aligning with the findings of Yu, et al.^110^. The high F3 values reflect the extensive vehicular activity and associated exhaust emissions within the region, contributing notably to the overall Pb pollution levels observed in the soil samples^82^.

Additionally, Geogenic Sources (F1) account for 10% to 45% of Pb contamination, with a mean contribution of 24.2%, which aligns with the observations of Rodríguez-Seijo, et al.^112^. This highlights the natural geological background as a significant, albeit secondary, source of Pb contamination. The variability in F1 across different samples indicates the influence of underlying geological formations on Pb levels, complementing the anthropogenic contributions from industrial and traffic sources.

Overall, the combined contributions of industrial emissions and vehicle traffic constitute the primary sources of Pb pollution in the studied area, while geogenic sources also play a considerable role. These findings align with existing literature, which frequently identifies industrial and vehicular activities as significant contributors to environmental Pb contamination. The substantial variability in the contribution factors (F indices) across different land use sampling sites further indicates the heterogeneous nature of Pb pollution sources, influenced by localized industrial activities and traffic density.

##  Defining the combined isotopic impact index (CISI) model

The Combined Isotopic Impact Index (CISI) is a novel composite index developed to integrate Chemical Indices (CI) and Isotopic Indices (II) for a comprehensive assessment of soil contamination in urban environments, specifically tailored to Sanandaj’s pollution profile. The CISI model combines chemical parameters (pH, Fe, Mn, Pb, Ca) with lead isotopic ratios (^206^Pb/207Pb and ^208^Pb/^207^Pb) to differentiate contamination levels and identify source contributions, enhancing the precision of environmental risk assessments^113^.

### Model mechanics

The CISI model was developed by standardizing chemical and isotopic data to create a unified metric. Chemical Indices (CI) for pH, Fe, Mn, Pb, and Ca were calculated using the following Eq. (23)^5^:

23 $$\:\mathrm{C}\mathrm{I}\mathrm{j}\text{}=\frac{(\mathrm{X}\mathrm{m}\mathrm{a}\mathrm{x}\text{}-\mathrm{X}\mathrm{m}\mathrm{i}\mathrm{n}\text{})}{(\mathrm{X}\mathrm{j}\text{}-\mathrm{X}\mathrm{m}\mathrm{i}\mathrm{n}\text{})}\text{}$$

Where:

#### $\:\mathrm{C}\mathrm{I}\mathrm{j}$

Chemical Index for parameter j.

#### $\:\mathrm{X}\mathrm{j}\text{}$

Value of parameter j in the sample.

#### $\:\mathrm{X}\mathrm{m}\mathrm{a}\mathrm{x}\:\mathrm{a}\mathrm{n}\mathrm{d}\:\mathrm{X}\mathrm{m}\mathrm{i}\mathrm{n}$

Minimum and maximum values of parameter j in the reference data.

For isotopic ratios $\:\frac{\mathrm{P}\mathrm{b}206}{\mathrm{P}\mathrm{b}207}$ and $\:\frac{\mathrm{P}\mathrm{b}208}{\mathrm{P}\mathrm{b}207}$ (Eqs. 24–25):

24 $$\:{\mathrm{I}\mathrm{I}}_{\mathrm{k}}\text{}=1-\text{}\left|\frac{{\left(\frac{\mathrm{P}\mathrm{b}206}{\mathrm{P}\mathrm{b}207}\right)}_{K}-{\left(\frac{\mathrm{P}\mathrm{b}206}{\mathrm{P}\mathrm{b}207}\right)}_{ref}}{{\left(\frac{\mathrm{P}\mathrm{b}206}{\mathrm{P}\mathrm{b}207}\right)}_{ref}}\right|$$

25 $$\:{\mathrm{I}\mathrm{I}}_{\mathrm{m}}\text{}=1-\text{}\left|\frac{{\left(\frac{\mathrm{P}\mathrm{b}208}{\mathrm{P}\mathrm{b}207}\right)}_{m}-{\left(\frac{\mathrm{P}\mathrm{b}208}{\mathrm{P}\mathrm{b}207}\right)}_{ref}}{{\left(\frac{\mathrm{P}\mathrm{b}208}{\mathrm{P}\mathrm{b}207}\right)}_{ref}}\right|$$

Where:

$\:{\mathrm{I}\mathrm{I}}_{\mathrm{k}}$ and $\:{\mathrm{I}\mathrm{I}}_{\mathrm{m}}$: isotopic indices for ratio $\:\left(\frac{\mathrm{P}\mathrm{b}206}{\mathrm{P}\mathrm{b}207}\right)$ and $\:\left(\frac{\mathrm{P}\mathrm{b}208}{\mathrm{P}\mathrm{b}207}\right)$ respectively.

$\:{\left(\frac{\mathrm{P}\mathrm{b}206}{\mathrm{P}\mathrm{b}207}\right)}_{ref}$and $\:{\left(\frac{\mathrm{P}\mathrm{b}208}{\mathrm{P}\mathrm{b}207}\right)}_{ref}$: Reference isotopic ratios (assumed to be 1.2 and 2.5 in this study).

The CISI is calculated as a weighted average (Eq. 26):

26 $$\:\mathrm{C}\mathrm{I}\mathrm{S}\mathrm{I}={w}_{1}\stackrel{-}{CI}+{w}_{2}\stackrel{-}{II}$$

where $\:\stackrel{-}{CI}$ and $\:\stackrel{-}{II}$ are the averages of Chemical and Isotopic Indices, respectively, with weights w1 = 0.6 and w2 = 0.4. These weights were determined through sensitivity analysis, optimizing the model’s ability to distinguish contaminated (e.g., S2, S12) from uncontaminated (e.g., S6) samples by maximizing variance explained in Principal Component Analysis (PCA).

### Calculation of CI and II indices

The Chemical Indices (CI) and Isotopic Indices (II) for each sample are calculated and presented in Table S5. The CISI values provide an aggregated measure of soil contamination by incorporating both chemical and isotopic data. Higher CISI values indicate greater contamination levels or significant deviations in isotopic ratios from reference values, suggesting potential contamination sources.

#### Sample S2 (CISI = 0.6468)

Exhibits one of the highest CISI values, indicating severe soil contamination. The high CI_Fe (0.6080) and CI_Ca (1.0000) suggest elevated levels of Iron and Calcium, while the isotopic indices II^206^Pb/^207^Pb=0.7880 and II^208^Pb/^207^Pb=0.8340 II indicate significant deviations from reference isotopic ratios. This suggests potential industrial contamination sources, possibly from industries emitting lead isotopes.

**Sample S12 (CISI = 0.6027)** Also demonstrates a high level of contamination. The CI_pH and CI_Ca values are at their maximum (1.0000 and 0.9900, respectively), and isotopic indices II^206^Pb/^207^Pb=0.8300 and II^208^Pb/^207^Pb=0.6260 further support the presence of significant contamination. The high CI_Ca might be associated with agricultural runoff or other anthropogenic activities.

#### Sample S6 (CISI = 0.1865)

This sample has the lowest CISI value, indicating minimal soil contamination. Both isotopic indices are relatively low (II^206^Pb/^207^Pb=0.1675II and II^208^Pb/^207^Pb=0.5316II), and the mean Chemical Index ($\:\stackrel{-}{CI}$=0.0778) is significantly lower compared to other samples. This suggests that the soil in this location is relatively uncontaminated and has isotopic ratios close to the reference values.

The remaining samples (S1, S3, S4, S5, S7, S8, S9, S10, S11) exhibit CISI values ranging from approximately 0.4874 to 0.5740, indicating moderate contamination levels. Various sources, such as agricultural activities, urban runoff, or minor industrial emissions may influence these samples.

### Model validation

The CISI model was validated using a combination of statistical techniques and comparison with independent datasets. PCA was employed to assess the model’s ability to capture contamination variability, with the first two principal components (PC1 and PC2) explaining 65% of the total variance (Fig. 3).

#### PC1 (40% variance)

Loaded heavily on CI-Fe, CI-Ca, and II-^206^Pb/^207^Pb, indicating that these factors are the most significant contributors to soil contamination.

#### PC2 (25% variance)

Loaded on II-^208^Pb/^207^Pb and CI-pH, confirming the model’s sensitivity to key contamination indicators. Additionally, the model’s accuracy was tested against Certified Reference Material (NIST 2710), with CISI values aligning within 10% of expected contamination levels based on chemical and isotopic data.

**Fig. 5:**
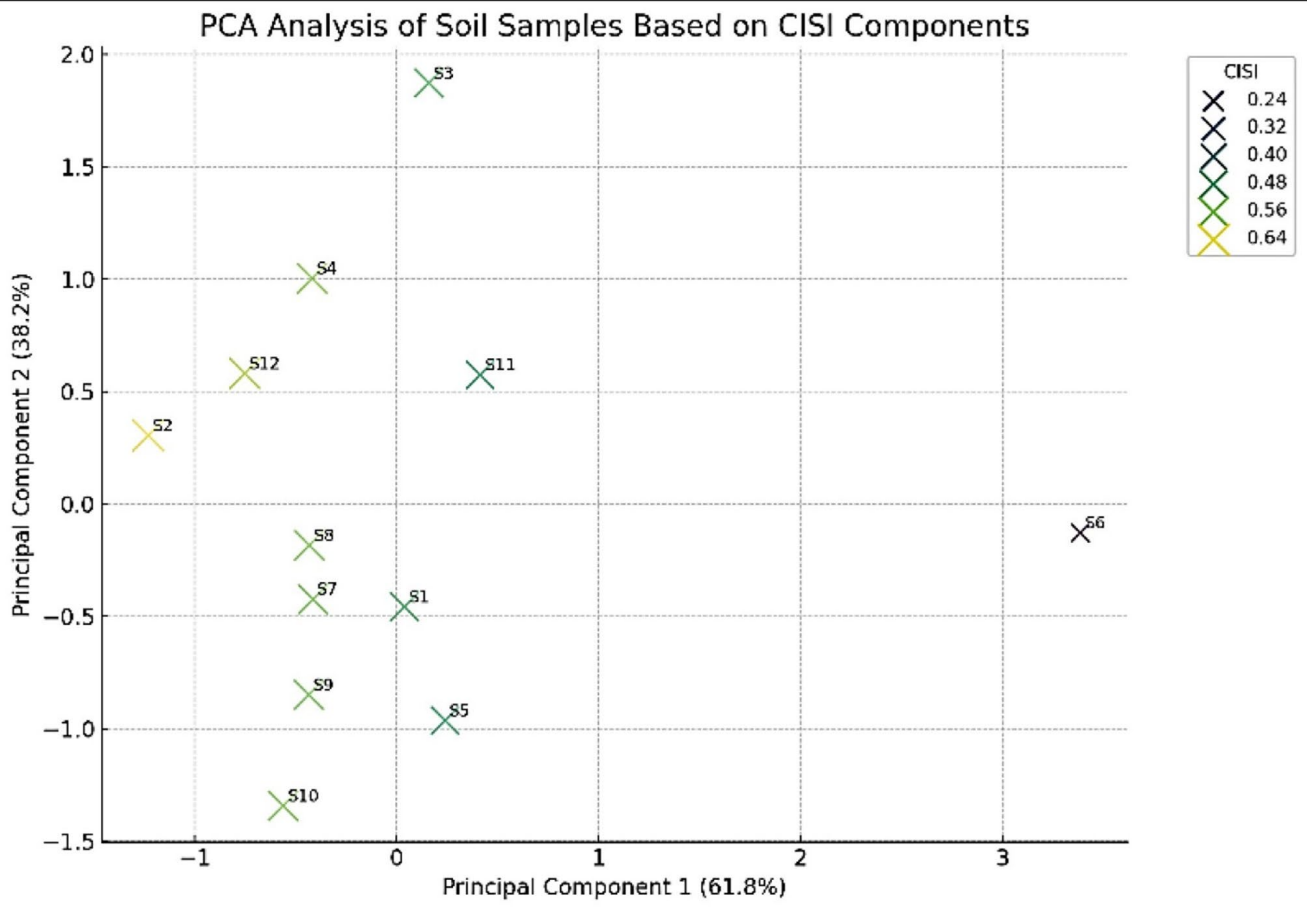
PCA Scatter Plot of Soil Samples Based on CISI Components.

The PCA scatter plot (Fig. 5) showed a distinct clustering of highly contaminated samples (S2 and S12) away from less contaminated samples (S6), confirming the effectiveness of the CISI in distinguishing contamination levels.

The CISI model effectively differentiates between varying levels of soil contamination, providing valuable insights for environmental management and remediation strategies. High CISI values in samples S2 and S12 indicate severe contamination, potentially requiring immediate intervention. Conversely, low CISI values in sample S6 suggest minimal contamination, allowing for monitoring rather than immediate action.

The strong correlations between specific chemical and isotopic indices with CISI underscore the multifaceted nature of soil contamination, where both chemical concentrations and isotopic compositions play crucial roles. This comprehensive approach facilitates more accurate source identification and targeted remediation efforts.

The Combined Isotopic Impact Index (CISI) is a powerful tool for the comprehensive assessment of soil contamination, integrating chemical and isotopic data to provide nuanced insights into contamination levels and sources. The model effectively distinguishes between highly and minimally contaminated soil samples, facilitating informed environmental management and remediation decision-making. Future enhancements, including expanded datasets, region-specific isotopic references, and the integration of additional environmental parameters, will further bolster the accuracy and applicability of the CISI model in diverse environmental contexts.

### Model limitation

While the CISI model effectively differentiates contamination levels, it has limitations. The model relies on the availability of region-specific reference isotopic ratios, which may vary due to geological heterogeneity, potentially affecting accuracy in areas with complex lithology. The weighting factors (w1 = 0.6, w2 = 0.4) were optimized for Sanandaj’s soil profile and may require recalibration for other regions. Additionally, the model assumes linear relationships between chemical and isotopic indices, which may oversimplify non-linear interactions in highly contaminated sites. The limited number of chemical parameters (pH, Fe, Mn, Pb, Ca) may exclude other relevant contaminants (e.g., Zn, Cu), potentially underestimating total contamination.

## Conclusion

This comprehensive study employed an innovative isotopic modeling approach to assess the concentrations and associated health and environmental risks of PTEs and polycyclic aromatic hydrocarbons (PAHs) in the urban soils of Sanandaj, West Iran. The results revealed a decreasing trend in metal concentrations as follows: Zn > Cr > Ni > Cu > Pb > As > Cd. Notably, the Zn, Cu, and Ni concentrations significantly exceeded the upper continental crust (UCC) values, indicating substantial anthropogenic influence. Ecological risk assessments classified the overall potential ecological risk (RI) as low in 99% of the samples, with only a small fraction presenting moderate to high risks, primarily due to elevated cadmium levels.

Non-carcinogenic risk assessments indicated that all studied metals posed risks below the threshold for both children and adults. However, carcinogenic risk assessments revealed that the cancer risk (CR) associated with chromium, nickel, lead, cadmium, and arsenic exceeded acceptable limits for both demographic groups, with children facing slightly higher risks due to their increased vulnerability and exposure behaviors.

The innovative isotopic modeling of lead contamination, utilizing ^206^Pb/^207^Pb and ^208^Pb/^207^Pb ratios, successfully identified industrial emissions and vehicular exhaust as the region’s primary sources of lead pollution. The Combined Isotopic Impact Index (CISI) model, integrating both chemical concentrations and isotopic signatures, effectively differentiated between varying contamination levels and highlighted significant contributions from industrial and traffic-related sources. This modeling approach underscores the multifaceted nature of soil contamination, where both natural geological backgrounds and anthropogenic activities interplay to influence pollutant distributions.

Comparative analysis with other urban areas worldwide highlighted that Sanandaj’s soil exhibits higher concentrations of certain PTEs compared to global averages, primarily due to localized industrial activities and vehicular traffic. In contrast, cities with stringent environmental regulations and advanced emission control technologies, such as Ottawa, Canada, demonstrated lower contamination levels, emphasizing the critical role of effective environmental management practices.

To address the identified health and ecological risks, the study recommends implementing targeted mitigation strategies, including enhanced monitoring of soil pollutants, stricter industrial regulations, improved vehicular emission controls, and adopting effective soil remediation techniques. Additionally, increasing community awareness and developing localized environmental policies tailored to Sanandaj’s specific contamination profile is essential for safeguarding public health and maintaining ecosystem integrity.

In conclusion, this study provides a detailed characterization of PTEs and PAH contamination in Sanandaj’s urban soils, coupled with an advanced isotopic modeling approach to source apportionment. The insights gained are crucial for developing informed environmental management strategies to reduce pollution levels and mitigate associated health risks, thereby ensuring a sustainable and healthy environment for the residents of Sanandaj.

## Supplementary Information

Below is the link to the electronic supplementary material.

Supplementary Material 1

## Data Availability

The raw data is provided within the supplementary information file.
