# Supplementary material for: Innovative isotopic modeling and risk assessment of PTEs and PAHs in urban soils
Source: Sci Rep. 2025 Nov 3;15:38380. doi: 10.1038/s41598-025-22183-z (PMC12583582; doi:10.1038/s41598-025-22183-z)
Supplement: Supplementary file 1 — Supplementary Material 1 [file 41598_2025_22183_MOESM1_ESM.docx]

Table S1: Recovery Rates and Precision for NIST 2710 Certified Reference Material (CRM)

| **Element** | **LOD**  **(mg/kg)** | **LOQ**  **(mg/kg)** | **Certified Concentration (mg/kg)** | **Measured Concentration (mg/kg)** | **Recovery Rate (%)** | **RSD (%) (n=3)** |
| --- | --- | --- | --- | --- | --- | --- |
| Chromium (Cr) | 0.04 | 0.13 | 100 ± 5 | 98.5 | 98.5 | 3.2 |
| Nickel (Ni) | 0.03 | 0.10 | 120 ± 5 | 118.7 | 98.9 | 2.7 |
| Copper (Cu) | 0.02 | 0.07 | 85 ± 5 | 83.2 | 97.9 | 4.1 |
| Lead (Pb) | 0.01 | 0.03 | 110 ± 5 | 107.4 | 97.8 | 3.5 |
| Zinc (Zn) | 0.05 | 0.17 | 150 ± 5 | 152.1 | 101.4 | 2.9 |

### Table Notes:

**Certified Concentration (mg/kg):** Certified concentrations from the NIST 2710 reference material.

**Measured Concentration (mg/kg):** Measured concentrations determined using ICP-MS.

**Recovery Rate (%):** Percentage recovery, indicating the accuracy of the measurements, expected to be in the range of 95% to 105%.

**RSD (%) (n=3):** Relative standard deviation for duplicates (3 replicates), demonstrating precision and reproducibility of the results.

**LOD (mg/kg):** Limit of detection, calculated as three times the standard deviation of 10 blank samples.

**LOQ (mg/kg):** Limit of quantification, calculated as 10 times the standard deviation of blank samples.

Table S2. parameters of the equations for evaluating the carcinogenic and non-carcinogenic risk of PTEs in surface soil

| **Factor** | **Unit** | **Adults** | **Children** |
| --- | --- | --- | --- |
| IngR | mg/day | 100 | 200 |
| InhR | m^3^/day | 12.8 | 7.63 |
| EF | day/year | 350 | 350 |
| ED | year | 24 | 6 |
| BW | kg | 55.9 | 15 |
| AT | days | ED*365 | ED*365 |
| PEF | m^3^/kg | 1.36 | 1.36 |
| SA | cm^2^ | 4350 | 1600 |
| AF | mf. Cm day | 0.7 | 0.2 |
| ABS | - | 0.001 | 0.001 |
| RfD_Ingestion_ | mg/kg-day | Cr (3.00E-03), Ni (2.00E-02), Cu (4.00E-02), Zn (3.00E-01), Cd (1.00E-03), Pb (3.00E-03), As (3.00E-04), Hg (3.00E-04), V (7.00E-03). | |
| RfD_Inhalation_ | mg/kg-day | Cr (2.86E-05), Ni (2.6E-04), Cu (4.02E-02), Zn (3.00E-01), Cd (1.00E-01), Pb (3.52E-03), As (3.1E-04), Hg (8.57E-05). | |
| RfD_Dermal_ | mg/kg-day | Cr (6.00E-05), Ni (5.4E-04), Cu (1.20E-04), Zn (6.00E-02), Cd (1.00E-05), Pb (5.25E-04), As (1.23E-04), Hg (2.10E-04), V (7.00E-05). | |

Table S3. Descriptive statistics of polycyclic aromatic compounds in surface soils of Sanandaj city

| Compound | Abv | ring | TEF | Mean | Max | Min | SDEV | Skewness |  |
| --- | --- | --- | --- | --- | --- | --- | --- | --- | --- |
| Naphthalene | Nap | 2 | 0.001 | 4.46 | 12.13 | 0.06 | 3.78 | 1 |  |
| Acenaphthylene | Acy | 3 | 0.001 | 9.2 | 23.38 | 2 | 5.15 | 1.44 |  |
| Acenaphthene | Ace | 3 | 0.001 | 2.25 | 12.1 | 0.57 | 2.8 | 3.21 |  |
| Fluorene | Flu | 3 | 0.001 | 27.53 | 67.37 | 4.52 | 20.46 | 1.13 |  |
| Phenanthrene | Phe | 3 | 0.001 | 100.85 | 234.31 | 2.79 | 62.86 | 0.31 |  |
| Anthracene | Ant | 3 | 0.01 | 141.02 | 476.58 | 35.77 | 126.53 | 1.89 |  |
| Fluoranthene | Flt | 4 | 0.001 | 21.89 | 85.29 | 6.45 | 20.02 | 2.36 |  |
| Pyrene | Pyr | 4 | 0.001 | 12.61 | 96.47 | 0.07 | 24.26 | 3.09 |  |
| Benz[a]anthracene | BaA | 4 | 0.1 | 60.7 | 162.16 | 7.17 | 46.09 | 1.08 |  |
| Benzo[b]fluoranthene | BbF | 5 | 0.1 | 365.51 | 1062.26 | 8.46 | 311.95 | 0.95 |  |
| Benzo[k]fluoranthene | BkF | 5 | 0.1 | 62.57 | 173.91 | 11.06 | 50.38 | 1.2 |  |
| Benzo[a]pyrene | BaP | 5 | 1 | 0 | 0 | 0 | 0 | 0 |  |
| Indenopyrene | InP | 6 | 0.1 | 19.57 | 19.57 | 19.57 | 0 | 0 |  |
| Dibenz[a,h]anthracene | DBA | 5 | 1 | 31.65 | 35.34 | 27.95 | 3.69 | 0 |  |
| Benzo[ghi]perylene | BghiP | 6 | 0.01 | 0 | 0 | 0 | 0 | 0 |  |
| 2 rings % |  |  |  | 0.52 | 0.49 | 0.05 | 0.56 | 1 |  |
| 3 rings % |  |  |  | 33.01 | 33.07 | 36.1 | 32.12 | 2.91 |  |
| 4 rings % |  |  |  | 11.19 | 13.98 | 10.83 | 13.33 | 1.87 |  |
| 5 rings % |  |  |  | 52.98 | 51.67 | 37.54 | 53.99 | 1.76 |  |
| 6 rings % |  |  |  | 2.3 | 0.8 | 15.48 | 0 | 0 |  |
| Σ15 PAH |  |  |  | 850.51 | 2460.87 | 126.44 | 677.95 | 17.66 |  |
| LMW PAHs |  |  |  | 285.31 | 825.87 | 45.71 | 221.57 | 8.68 |  |
| HMW PAHs |  |  |  | 566.5 | 1635 | 80.73 | 456.38 | 8.99 |  |
| COMPAHs/ΣPAHs |  |  |  | 0.76 | 0.74 | 0.42 | 0.77 | 0.5 |  |
| CANPAHs/ΣPAHs |  |  |  | 0.56 | 0.57 | 0.21 | 0.6 | 0.18 |  |
| NCANPAHs/ΣPAHs |  |  |  | 0.38 | 0.41 | 0.4 | 0.39 | 0.82 |  |
| TEQ |  |  |  | 83.17 | 182.43 | 32.95 | 45.94 | 0.35 |  |
| TEQ/ΣPAHs |  |  |  | 10% | 0.07 | 0.26 | 0.07 | 0.02 |  |

ΣPAHs: Sum of individual mass content of detected PAHs.

LMWPAHs: Sum of low molecular weight (3 ring) PAHs.

HMWPAHs: Sum of high molecular weight (4–6 ring) PAHs.

COMPAHs: Sum of major combustion-derived PAH content.

CANPAHs: Sum of carcinogenic PAHs.

NCANPAHs: Sum of non-carcinogenic PAHs.

TEF: Toxic equivalency factors

TEQ: Toxic equivalency concentration.

Table S4. Percent of each possible sources of Pb in the surface soil samples of Sanandaj city.

|  | Sample No. | pH | Fe (gr/kg) | Mn (gr/kg) | Pb (mg/kg) | Cd  (mg/kg) | ^204^Pb | ^206^Pb | ^207^Pb | ^208^Pb |
| --- | --- | --- | --- | --- | --- | --- | --- | --- | --- | --- |
| Industrial Area | S1 | 7.9 | 97.58 | 4.25 | 189 | 5.64 | 1.35 | 4.324 | 4.3841 | 7.548 |
|  | S2 | 7.8 | 114.6 | 3.28 | 245 | 4.58 | 1.87 | 3.487 | 3.6841 | 7.685 |
|  | S3 | 8.5 | 81.68 | 4.26 | 341 | 6.54 | 1.45 | 4.518 | 6.5487 | 6.457 |
|  | S4 | 8.4 | 176.45 | 2.38 | 284 | 7.84 | 1.32 | 3.967 | 4.3257 | 5.685 |
| Parks | S5 | 7.6 | 44.58 | 3.24 | 328 | 3.28 | 0.45 | 4.571 | 3.6847 | 5.624 |
|  | S6 | 7.9 | 31.58 | 1.68 | 128 | 2.67 | 0.45 | 0.654 | 3.2547 | 4.325 |
| Traffic Area | S7 | 8.7 | 45.68 | 1.58 | 237 | 1.37 | 1.34 | 3.418 | 2.6954 | 7.651 |
|  | S8 | 7.8 | 41.3 | 5.64 | 125 | 4.39 | 0.84 | 3.784 | 3.6584 | 6.324 |
|  | S9 | 8.4 | 61.5 | 4.39 | 175 | 2.84 | 0.98 | 3.514 | 2.5847 | 7.521 |
|  | S10 | 8.6 | 35.4 | 1.84 | 245 | 6.54 | 1.23 | 2.947 | 2.6952 | 6.252 |
| Residential | S11 | 8.6 | 29.54 | 6.54 | 143 | 2.14 | 0.89 | 2.957 | 3.4751 | 4.251 |
|  | S12 | 8.7 | 18.64 | 3.57 | 169 | 4.54 | 0.72 | 2.684 | 2.6954 | 4.2154 |

Table S5. Chemical Indices (CI) and Isotopic Indices (II) for each sample

| Sample No. | CI_pH | CI_Fe | CI_Mn | CI_Pb | CI_Ca | Mean_CI | II-^206^Pb/207Pb | II-^208^Pb/^207^Pb | Mean_II | CISI |
| --- | --- | --- | --- | --- | --- | --- | --- | --- | --- | --- |
| S1 | 0.272 | 0.501 | 0.690 | 0.296 | 0.003 | 0.352 | 0.822 | 0.688 | 0.755 | 0.513 |
| S2 | 0.181 | 0.608 | 0.342 | 0.555 | 1 | 0.537 | 0.788 | 0.834 | 0.811 | 0.646 |
| S3 | 0.818 | 0.4 | 0.540 | 1 | 0.009 | 0.553 | 0.575 | 0.394 | 0.484 | 0.526 |
| S4 | 0.727 | 1 | 0.161 | 0.735 | 0.012 | 0.527 | 0.763 | 0.525 | 0.644 | 0.574 |
| S5 | 0 | 0.164 | 0.334 | 0.939 | 0.003 | 0.288 | 0.966 | 0.610 | 0.788 | 0.488 |
| S6 | 0.272 | 0.082 | 0.020 | 0.013 | 0.001 | 0.077 | 0.167 | 0.531 | 0.349 | 0.186 |
| S7 | 1 | 0.171 | 0 | 0.518 | 0.295 | 0.396 | 0.773 | 0.83 | 0.801 | 0.558 |
| S8 | 0.181 | 0.143 | 0.819 | 0 | 0.957 | 0.420 | 0.862 | 0.692 | 0.777 | 0.563 |
| S9 | 0.727 | 0.271 | 0.567 | 0.231 | 0.001 | 0.36 | 0.866 | 0.835 | 0.850 | 0.556 |
| S10 | 0.909 | 0.106 | 0.052 | 0.555 | 0.009 | 0.326 | 0.911 | 0.927 | 0.919 | 0.563 |
| S11 | 0.909 | 0.069 | 1 | 0.083 | 0 | 0.412 | 0.71 | 0.49 | 0.6 | 0.487 |
| S12 | 1 | 0 | 0.401 | 0.203 | 0.99 | 0.519 | 0.83 | 0.626 | 0.728 | 0.602 |
